# A novel molecular classification system based on the molecular feature score identifies patients sensitive to immune therapy and target therapy

**DOI:** 10.3389/fimmu.2024.1466069

**Published:** 2024-11-26

**Authors:** Yang Li, Yinan Ding, Jinghao Wang, Xiaoxia Wu, Dinghu Zhang, Han Zhou, Pengfei Zhang, Guoliang Shao

**Affiliations:** ^1^ Zhejiang Cancer Hospital, Hangzhou Institute of Medicine (HIM), Chinese Academy of Sciences, Hangzhou, Zhejiang, China; ^2^ Clinical Research Center of Hepatobiliary and Pancreatic Diseases of Zhejiang Province, Hangzhou, Zhejiang, China; ^3^ School of Chemistry and Materials Science, University of Science and Technology of China, Hefei, Anhui, China; ^4^ School of Molecular Medicine, Hangzhou Institute for Advanced Study, University of Chinese Academy of Sciences, Hangzhou, Zhejiang, China; ^5^ University of Chinese Academy of Sciences, Beijing, China; ^6^ Institutes of Biomedical Sciences, Inner Mongolia University, Hohhot, China

**Keywords:** hepatocellular carcinoma, precision medicine, molecular classifications, prognostic nomogram, multidimensional feature scores, immune therapy, target therapy

## Abstract

**Background:**

Hepatocellular carcinoma (HCC) is heterogeneous and refractory with multidimensional features. This study aims to investigate its molecular classifications based on multidimensional molecular features scores (FSs) and support classification-guided precision medicine.

**Methods:**

Data of bulk RNA sequencing, single nucleotide variation, and single-cell RNA sequencing were collected. Feature scores (FSs) from hallmark pathways, regulatory cell death pathways, metabolism pathways, stemness index, immune scores, estimate scores, etc. were evaluated and screened. Then, the unsupervised clustering on the core FSs was performed and the characteristics of the resulting clusters were identified. Subsequently, machine learning algorithms were used to predict the classifications and prognoses. Additionally, the sensitivity to immune therapy and biological roles of classification-related prognostic genes were also evaluated.

**Results:**

We identified four clusters with distinct characteristics. C1 is characterized by high TP53 mutations, immune suppression, and metabolic downregulation, with notable responsiveness to anti-PD1 therapy. C2 exhibited high tumor purity and metabolic activity, moderate TP53 mutations, and cold immunity. C3 represented an early phase with the most favorable prognosis, lower stemness and tumor mutations, upregulated stroma, and hypermetabolism. C4 represented a late phase with the poorest prognosis, highest stemness, higher TP53 mutations, cold immunity, and metabolic downregulation. We further developed practical software for prediction with good performance in the external validation. Additionally, FTCD was identified as a classification-specific prognostic gene with tumor-suppressing role and potential as a therapeutic target, particularly for C1 and C4 patients.

**Conclusions:**

The four-layer classification scheme enhances the understanding of HCC heterogeneity, and we also provide robust predictive software for predicting classifications and prognoses. Notably, C1 is more sensitive to anti-PD1 therapies and FTCD is a promising therapeutic target, particularly for C1 and C4. These findings provide new insights into classification-guided precision medicine.

## 1 Introduction

Primary liver cancer remains a global health challenge, leading to 905,677 new cases and 830,180 deaths worldwide in 2020, especially a major threat to Asia and Africa (https://gco.iarc.fr) (1). The majority of primary liver cancers are hepatocellular carcinomas (HCC) (2). The major factors that contribute to the onset and progression of HCC include chronic viral hepatitis, alcohol intake, non-alcoholic fatty liver disease, and exposure to aflatoxins (3, 4). The appropriate therapies for HCC depend on the tumor stage, hepatic functional reserve, and the general condition of the patients. For patients with early-stage HCC, the curative therapies include surgical resection, liver transplantation, or radiofrequency ablation. Intermediate-stage HCC is typically managed with transarterial chemoembolization and radiation therapy, either as monotherapy or in combination with systemic therapies. Advanced-stage HCC requires systemic therapies, including traditional chemotherapy and emerging precision medicine approaches such as immune checkpoint inhibitors or molecular targeted therapies (5, 6). Despite these treatments, HCC remains a highly refractory disease with approximately 70% of the 5-year recurrence rate after curative therapies and a 5-year overall survival (OS) of less than 15% (7, 8).

Given the heterogeneity of HCC, understanding its molecular characteristics and classifications is essential for precise clinical management. So far, several typing schemes for HCC have been proposed, yet they typically rely on specific dimensions (9–12), or are tailored to particular subtypes, such as HBV-related HCC (13). Moreover, few schemes performed validation with external cohorts and provided user-friendly software. These factors restrict their ability to comprehensively capture the complex features of tumors and impede practical implementation in clinical settings.

To make out the molecular features of HCC comprehensively, we conducted evaluations and classifications for HCC based on multidimensional feature scores (FSs). These FSs included 50 hallmark pathways from the Molecular Signatures Database (MSigDB, https://www.gsea-msigdb.org/gsea/msigdb) (14), 7 regulatory cell death pathways (15–20), immune cell infiltration (21), mRNA stemness index (mRNAsi) (22), estimate score (23), stromal score, tumor purity, immune score, immune checkpoint score (24), tumor immune dysfunction and exclusion (TIDE) score (25–27), and a merged metabolic pathway from the MSigDB database. We also systematically characterized the response to anti-PD1 therapy for each subtype, as well as their single nucleotide variation (SNV), tumor immune microenvironment (TIME), drug sensitivity, pseudo-temporal trajectory, intercellular communication, etc. In this study, we also developed practical software for predicting the molecular subtypes and prognosis based on individual transcriptomic data (https://github.com/OliveryYL/oncoClassSurv). In addition, our study identified formiminotransferase cyclodeaminase (FTCD) as a classification-specific prognostic gene with anti-tumor effects in HCC, serving as a potential therapeutic target for specific molecular classifications through bioinformatics screening and experimental evidence.

**Figure 1:**
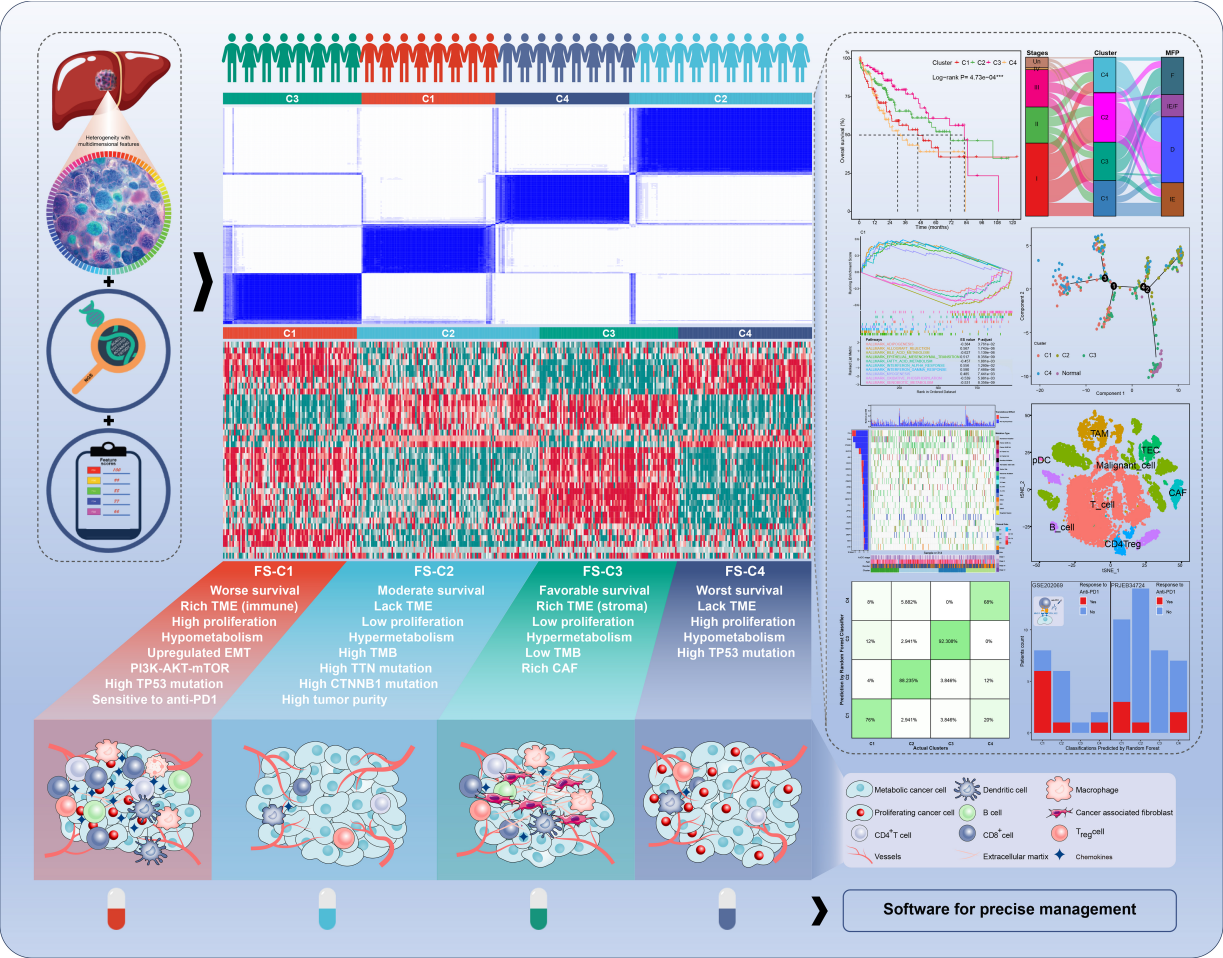
The graphic overview of the molecular classification system in the current study.

Therefore, this study provides a comprehensive global landscape of HCC from multiple perspectives, which can provide support to therapeutic decision-making based on classifications, individualized disease evaluation, and further scientific research (**Figure 1**).

## 2 Materials and methods

### 2.1 Research scheme

**Figure 2:**
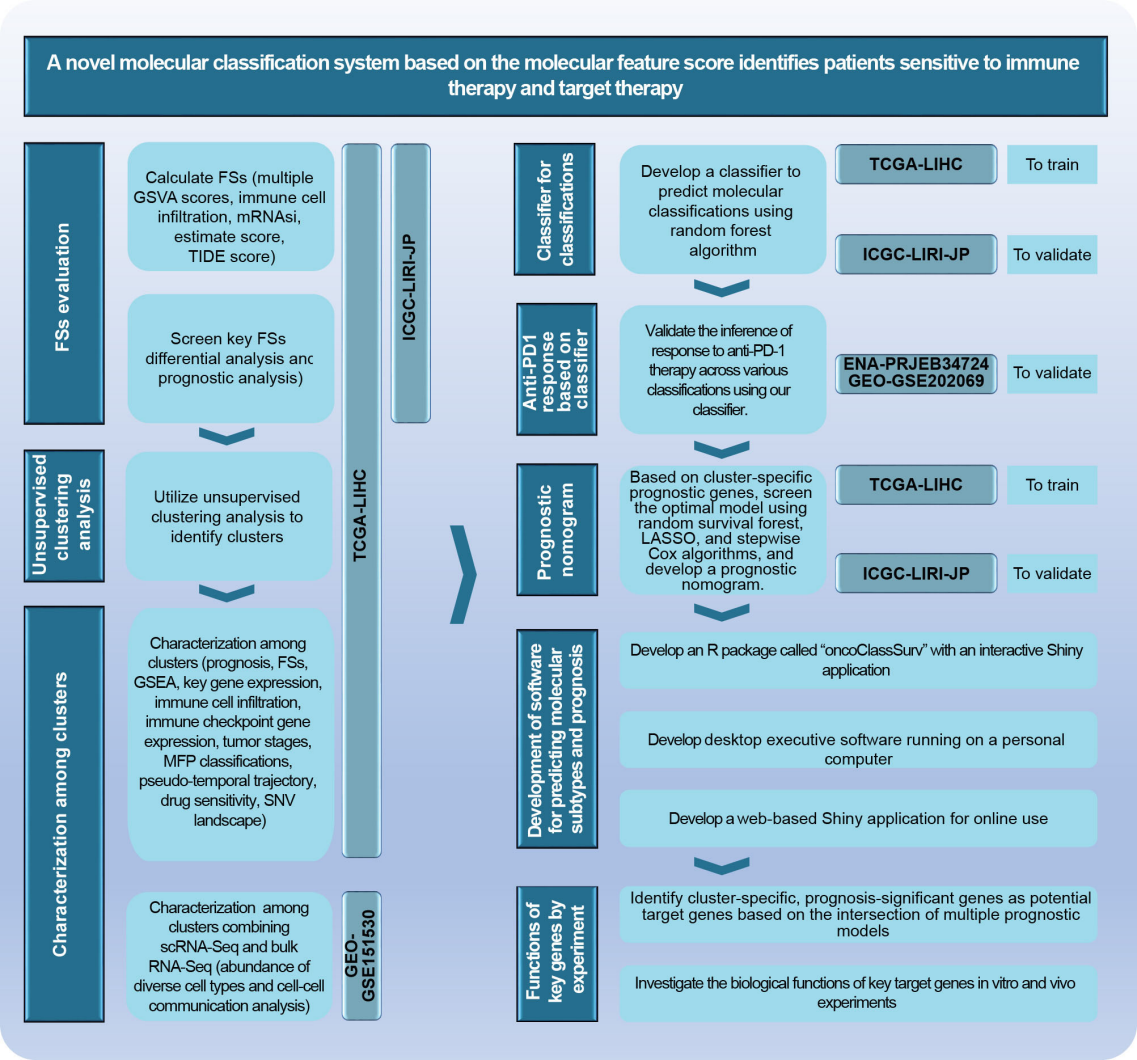
The main analytical scheme of the current study.

The research scheme is illustrated in **Figure 2**. Further details on analytic processes and experimental methods are provided in the **Supplementary Materials**.

### 2.2 Data collection and feature score calculation

Bulk RNA sequencing (RNA-Seq) data were obtained from the Cancer Genome Atlas-Liver Hepatocellular Carcinoma (TCGA-LIHC) and the International Cancer Genome Consortium (ICGC-LIRI-JP) projects. A total of 89 feature scores (FSs) were calculated, including gene set variation analysis (GSVA) scores (28), curated scores, and immune cell infiltration metrics. GSVA scores encompassed 50 hallmark gene sets and another 11 crucial gene sets (**Supplementary Table S1**). Six curated scores—mRNAsi, TIDE, estimate score, immune score, stromal score, and tumor purity—were calculated based on previous studies (22, 25, 26). Immune cell infiltration was estimated using the R package “immunedeconv”, which involves six algorithms: Cibersort, Quantiseq, Timer, MCPcounter, XCell, and EPIC (21). Specifically, the proportions of 22 immune cell types calculated by the Cibersort algorithm were enrolled in the 89 FSs above.

### 2.3 Clustering and characterization

Differential, prognostic, and Venn analyses were performed to identify consistently significant FSs in HCC. These FSs were then used for the unsupervised clustering analysis (29). The characteristics of clusters were analyzed, including key signal pathways, immune evaluation, pseudo-temporal trajectory, single nucleotide variation (SNV), drug sensitivity, and single-cell RNA sequencing (scRNA-Seq). Moreover, to make out the association among diverse classification criteria, we compared the proportion of different stages and MFP subtypes among clusters, respectively (30).

### 2.4 Classifier development and validation

Cluster-specific marker genes were identified employing the “FindAllMarkers” function from the “Seurat” package (31). A classifier was developed using the random forest (RF) algorithm based on these marker genes in the TCGA-LIHC training cohort and externally validated with the ICGC-LIRI-JP cohort. To validate the reliability of our classifier, characteristics of the predicted classifications in the external cohort were compared with those of the clusters in the TCGA-LIHC cohort. Additionally, datasets GSE202069 and PRJEB34724 were used to validate the responses to anti-PD1 therapy based on our classifier (12, 32, 33).

### 2.5 Prognostic nomogram

A novel prognostic nomogram was developed using cluster-specific marker genes in the TCGA-LIHC training cohort and externally validated with the ICGC-LIRI-JP cohort.

### 2.6 Software development

The practical software was developed for individualized predictions of molecular classifications and prognoses using external transcriptomic data.

### 2.7 Classification-related therapeutic target identification

Significant therapeutic targets were identified through Venn analysis, differential expression analysis, and prognostic analysis of genes from several prognostic models. Lentiviral transduction was used to establish overexpressing Huh7 cell lines for the target gene and its negative control. The biological roles of the gene were evaluated by means of cell proliferation, colony formation, transwell migration, apoptosis, cell cycle assays, and tumorigenicity assays in nude mice.

All animal procedures were approved by the Ethics Committee of Zhejiang Cancer Hospital and conducted in accordance with the ethical guidelines.

## 3 Results

### 3.1 Identification for critical FSs and unsupervised clustering analysis.

**Figure 3:**
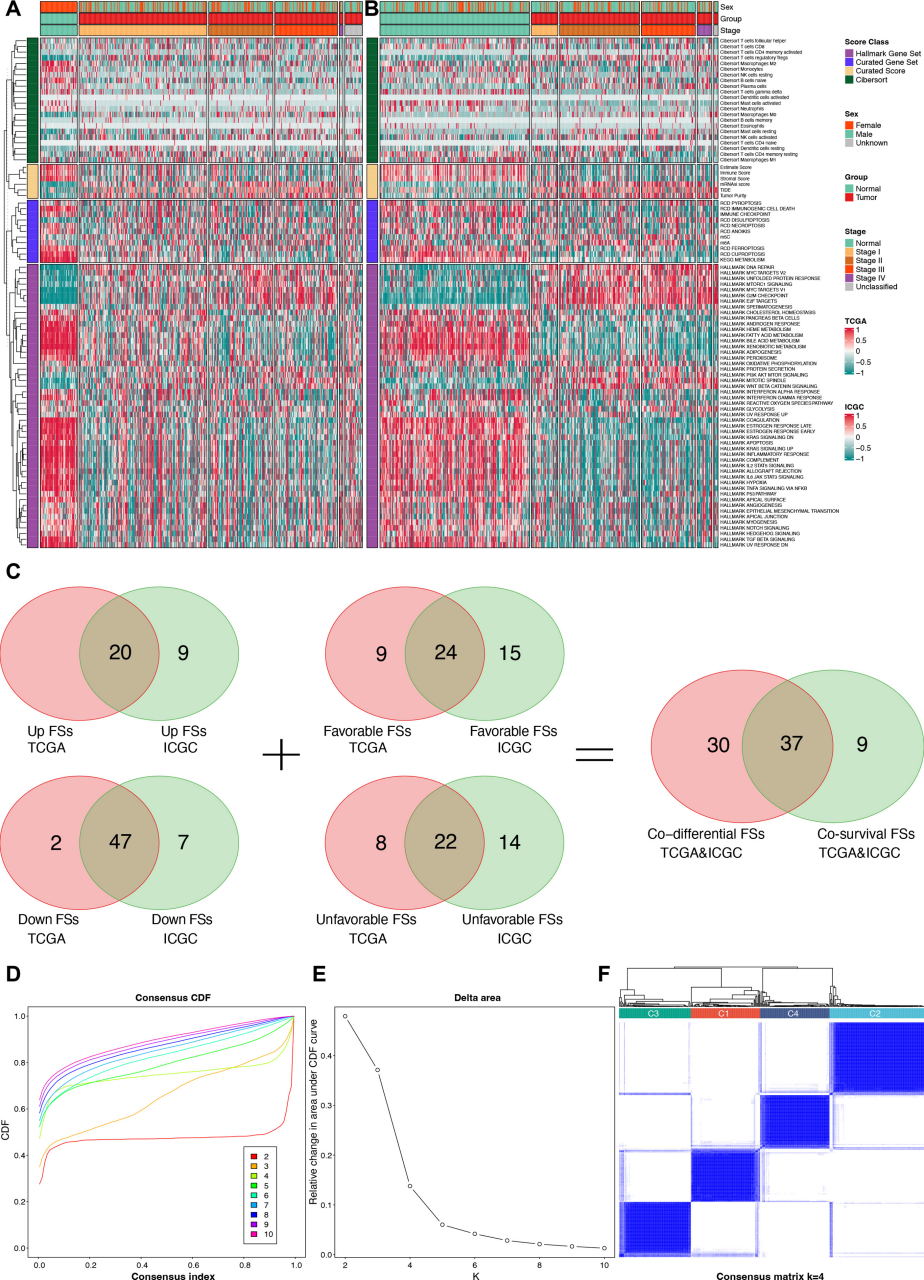
Identification for critical FSs and unsupervised clustering analysis. **(A)** Differential heat map of FSs in the TCGA cohort. **(B)** Differential heat map of FSs in the ICGC cohort. **(C)** Selection of consistently differential and prognostic FSs using Venn analyses. **(D)** The consensus CDF plot of the unsupervised clustering analysis. **(E)** The delta area plot of the unsupervised clustering analysis. **(F)** Consensus matrix plot of the unsupervised clustering analysis when K = 4. FSs, feature scores; TCGA, the Cancer Genome Atlas; ICGC, the International Cancer Genome Consortium; CDF, consensus cumulative distribution function.

After calculating the 89 FSs, differential and prognostic analyses were performed to identify the most critical FSs in the TCGA and the ICGC cohorts. The differential analysis results are presented in the heat maps (**Figures 3A, B**) and **Supplementary Table S2**, while the Kaplan-Meier survival analysis results are provided in **Supplementary Table S3**. The forest plots of the univariable Cox regression analyses are shown in **Supplementary Figure S1**. The results of the Kaplan-Meier survival analysis were incorporated into the subsequent screening. Finally, Venn analysis for the differential and the prognostic FSs identified 37 critical FSs closely related to HCC biological behavior (**Figure 3C**).

Using the 37 critical FSs, we performed unsupervised clustering analysis on 374 tumor samples from the TCGA-LIHC cohort, identifying four clusters: C1, C2, C3, and C4 (**Figures 3D–F**).

### 3.2 Prognostic characteristics among clusters

**Figure 4:**
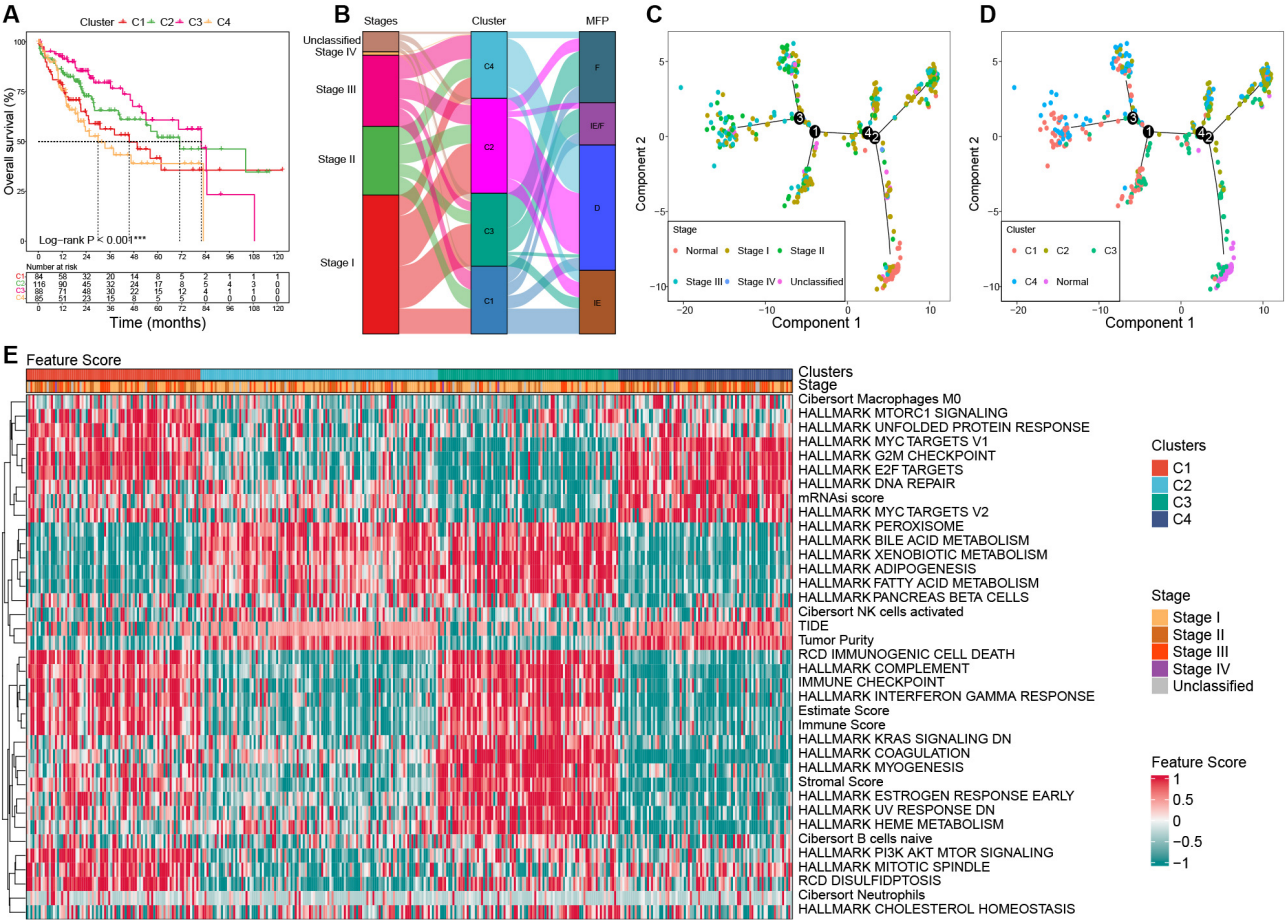
Characterization among clusters based on bulk RNA-seq in the TCGA cohort. **(A)** Survival curves among clusters. **(B)** Association among clusters, stages, and MFP classifications using Sanky plot. **(C)** Pseudo-temporal trajectory analyses by tumor stages. **(D)** Pseudo-temporal trajectory analyses by clusters. **(E)** Heat map of the key FSs enrolled in the unsupervised clustering analysis among clusters in the TCGA cohort. TCGA, the Cancer Genome Atlas; MFP, molecular functional portrait.

Significant survival differences were observed across the clusters. Patients in C3 had the most favorable prognosis, with a median overall survival (OS) of 81.9 months, followed by C2 (median OS: 71 months), C1 (median OS: 45.7 months), and C4 (median OS: 30 months) (*P* < 0.001, **Figure 4A**).

### 3.3 Association among clusters, tumor stages, and MFP classifications

The results showed that C3 had the highest proportion of stage I tumors (58.14%) and the lowest proportion of stage III (12.79%), while C4 had the highest proportion of stage III tumors (35.44%) and the lowest proportion of stage I (31.65%). C1 was predominantly MFP-IE (37.50%), whereas C2 and C4 were mainly MFP-D (64.29% and 72.15%, respectively). C3 showed the highest frequency of MFP-F (47.67%) (**Figure 4B**).

### 3.4 Pseudo-temporal trajectory analysis

To investigate the evolutionary relationships among clusters, we performed a pseudo-temporal trajectory analysis. As illustrated in **Figure 4C**, the lower-right subpopulation, representing normal samples, was designated as the starting point, while the top-left subpopulation marked the endpoint. The analysis revealed that cluster C3 was closest to normal tissue, followed by C2, C1, and C4, which aligns closely with their prognostic characteristics (**Figure 4D**).

### 3.5 Characteristics of key gene expression and FSs among clusters

Significant differences in gene expression and FSs were observed among clusters. The expression of MKI67 and AFP was significantly upregulated in C1 and C4 than that of C2 and C3. Compared to the normal samples, samples with upregulated AFP in HCC tumors mostly occurred in C1 and C4, not C2 and C3 (**Supplementary Figure S2**; **Supplementary Table S4**). The significant feature scores (FSs) in the heat map can be mainly categorized into three parts: stemness proliferation, microenvironment (immune/stroma), and metabolism. In terms of stemness proliferation, notable FSs include the mRNAsi score, MYC targets, E2F targets, G2M checkpoint, PI3K AKT mTOR pathway, and DNA repair activity. The microenvironment category encompasses the estimate score, stromal score, immune checkpoint score, immune score, complement score, immunogenic cell death score, tumor purity, and TIDE score. The metabolism category includes various metabolic-related pathway scores. These categories help to delineate the roles and impacts of FSs within the context of tumor biology.

C1 was characterized by upregulated activities in MYC targets, E2F targets, G2M checkpoint, and PI3K AKT mTOR. In addition, C1 had a high estimate score, hot immunity (such as immune checkpoint score, immune score, complement score, and immunogenic cell death score), and high disulfidptosis, while its tumor purity and TIDE score were low, as well as hypometabolism; C2 was characterized by downregulated PI3K AKT mTOR activity, cold immunity, high tumor purity, and hypermetabolism; C3 exhibited the lowest stemness, high estimate score and stromal score, hot immunity, low tumor purity, low DNA repair activity, and hypermetabolism; C4 showed the highest stemness, high G2M, high DNA repair, but low PI3K AKT mTOR activity, cold immunity, low estimate score and stromal score, high tumor purity, and hypometabolism (**Figure 4E**; **Supplementary Table S5**).

### 3.6 Gene set enrichment analysis

GSEA revealed significant pathway alterations among the clusters. In C1, there was upregulation in pathways such as epithelial-mesenchymal transition (EMT), interferon response, and antigen processing, while pathways like adipogenesis, fatty acid metabolism, and oxidative phosphorylation were downregulated. Conversely, C2 exhibited the opposite trends in these pathways compared to C1. C3 showed upregulation in EMT, interferon response, coagulation, and complement pathways, with downregulation observed in the unfolded protein response, G2M checkpoint, E2F targets, and MYC targets. In C4, significant upregulation was observed in the G2M checkpoint, E2F targets, ribosome, and oxidative phosphorylation pathways, while its peroxisome, immune, and metabolic pathways were significantly downregulated (**Supplementary Figure S3**; **Supplementary Table S6**).

### 3.7 Characteristics of immune microenvironment among clusters

We characterized the immune landscape among the four clusters to infer their responses to immunotherapy. Immune and stromal cell infiltration was evaluated using six computational methods: XCell, MCPcounter, Timer, Quantiseq, EPIC, and Cibersort (**Supplementary Figure S4A**). Overall, clusters C1 and C3 had higher levels of immune and stromal cells compared to C2 and C4. Notably, C1 exhibited the highest abundance of CD8+ T cells and the highest cytotoxicity score, indicating a strong anti-tumor immune response. C3 showed the highest abundance of cancer-associated fibroblasts (CAF) and endothelial cells, suggesting a tumor-promoting microenvironment. In addition, C1 and C3 had the highest abundance of myeloid dendritic cells and macrophages, which have complex roles in antigen presentation and tumor progression.

Further investigation into the expression of human leukocyte antigen (HLA) genes and immune checkpoint genes (ICGs) revealed significant upregulation of multiple HLA genes and ICGs in C1, including HLA-A, HLA-B, HLA-C, PDCD1, CD274, CTLA-4, HAVCR2, LAG3, CD80, CD86, TIGIT, etc. In contrast, although C3 had upregulated expression of HLA genes, its ICGs expression was weak (**Supplementary Figure S4B**).

These findings indicate that C1 is in an immunosuppressive state, potentially making it more responsive to immune checkpoint blockade (ICB) therapies by alleviating this inhibition and thereby inducing an immune response.

### 3.8 Single nucleotide variation landscape among clusters

Considering that SNV can drive tumor progression by altering the structure of encoded proteins or regulating gene expression, therefore, we investigated the SNV landscape among clusters.

**Figure 5:**
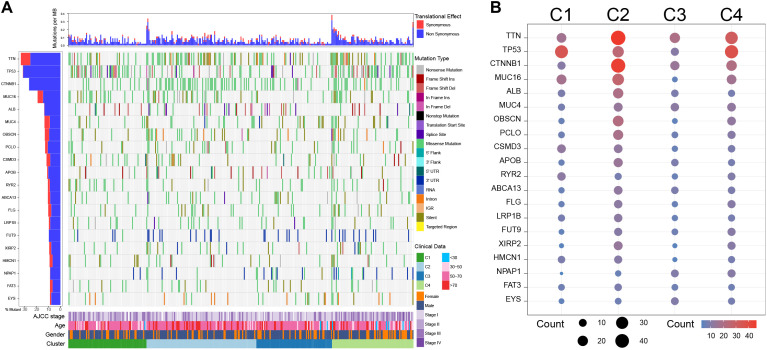
Single nucleotide variation analysis among clusters in the TCGA cohort. **(A)** Single nucleotide variation landscape among clusters. **(B)** Single nucleotide variation frequency among clusters.

The SNV analysis showed that C1 exhibited high-frequency TP53 missense mutations (29.58%), low frequencies of TTN (15.49%) and CTNNB1 (15.49%) missense mutations; C2 harbored multiple high-frequency mutations, especially TTN (28.00% missense mutations and 13.00% silent mutations) and CTNNB1 (43.00% of missense mutations), with moderate TP53 mutations (13.00% of missense mutations); Notably, C3 had the lowest overall mutations among the four clusters; C4 was mainly characterized by high frequencies of TP53 (33.78% missense mutations) and TTN (25.68% missense mutations) mutations, while the frequency of CTNNB1 mutations was not significantly increased (27.03% missense mutations) (**Figure 5**).

### 3.9 Drug sensitivity evaluation among clusters

To evaluate the differences in drug sensitivity among clusters, we calculated the half-maximal inhibitory concentration values for 198 drugs. The results revealed distinct drug sensitivities for each cluster. C1 exhibited increased sensitivity to several drugs: gemcitabine, which interferes with DNA synthesis; cisplatin, which induces DNA cross-linking and damage; irinotecan, which inhibits topoisomerase I; and talazoparib, which inhibits poly ADP ribose polymerase (PARP). C2 was primarily sensitive to TAF1 inhibitor TAF1_5496_1732, which inhibits transcription; MCL-1 inhibitor AZD5991_1720, which promotes apoptosis; and mitochondrial inhibitor Dihydrorotenone_1827. C3 showed heightened sensitivity to nutlin-3a, which inhibits MDM2; selumetinib, which inhibits MEK1/2; mitoxantrone, which inhibits topoisomerase II, and gemcitabine. C4 was sensitive to Sepatronium bromide_1941, which inhibits survivin; Daporinad_1248, which inhibits nicotinamide phosphoribosyltransferase (NAMPT); and MK−1775_1179, which inhibits WEE1 and PLK1 in the cell cycle pathway (**Supplementary Figure S5A**; **Supplementary Table S7**).

We further investigated the correlation between drug sensitivity and TP53 mutations. The results showed that the expression of TP53 and MDM2 was higher in wild-type TP53 patients compared to those with TP53 mutations. Most patients in the C3 subtypes met the conditions of wild-type TP53 and high expression of MDM2, which could explain the sensitivity of nutlin-3a in C3 patients (**Supplementary Figures S5B, C**).

### 3.10 Single-cell analysis for characterization among clusters

**Figure 6:**
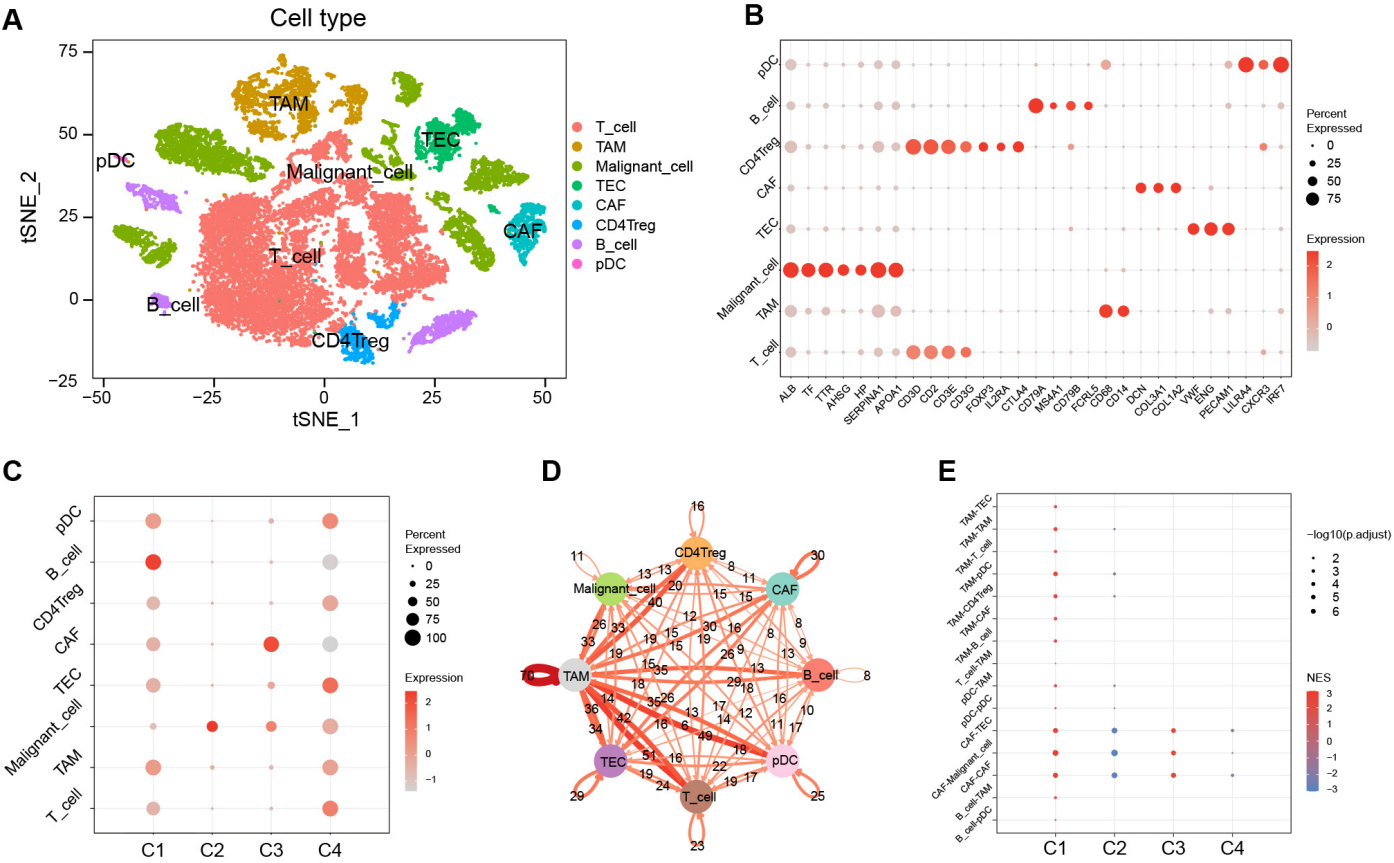
Characterization among clusters using scRNA-Seq data. **(A)** The tSNE plots of cell annotation for single-cell clusters. **(B)** Single-cell subpopulations and their annotation genes. **(C)** GSVA scores of upregulated marker gene sets of C1–C4 among the single-cell subpopulations. **(D)** Network of intercellular communication among single-cell subpopulations. **(E)** The activation level of intercellular communications among clusters in the TCGA cohort using the GSEA algorithm. tSNE, t-distributed stochastic neighbor embedding; GSVA, gene set variation analysis; TCGA, the Cancer Genome Atlas; GSEA, gene set enrichment analysis; NES, normalized enrichment score.

To gain deeper insights into clusters at the cell-subpopulation level, we performed single-cell analyses. Following quality control, dimensionality reduction, clustering, and cell annotation, we identified eight distinct cell subpopulations (**Supplementary Figure S6**; **Figures 6A, B**).

We evaluated the significantly upregulated marker gene sets of C1–C4 using the GSVA algorithm in single cells (**Figure 6C**). The results showed that the activity of the characteristic gene set of C1 was upregulated in various immune cell types, yet exhibited downregulated activity in malignant cells. In contrast, the activity of the characteristic gene set of C2 was significantly upregulated in malignant cells. The characteristic gene set of C3 showed pronounced activation in CAF, whereas the C4 characteristic gene set displayed activation across various cell types, predominantly in TEC, but showed diminished activity in B cells and CAF.

Cell-cell communication analysis revealed that tumor-associated macrophages (TAM) played a pivotal role in intercellular interactions within HCC (**Figure 6D**). The intercellular-interaction gene sets were used as reference gene sets for the GSEA enrichment analysis of C1–C4 based on bulk RNA-seq clustering (**Figure 6E**). The results showed upregulations of TAM-mediated and CAF-mediated intercellular communication in C1, a downregulation of CAF-mediated intercellular communication in C2, and an upregulation of intercellular communication mediated by CAF in C3 (*P* < 0.05). However, there was no significant enrichment of intercellular communication observed in C4.

### 3.11 Development of the classifier and validation for response to anti-PD1 therapy

**Figure 7:**
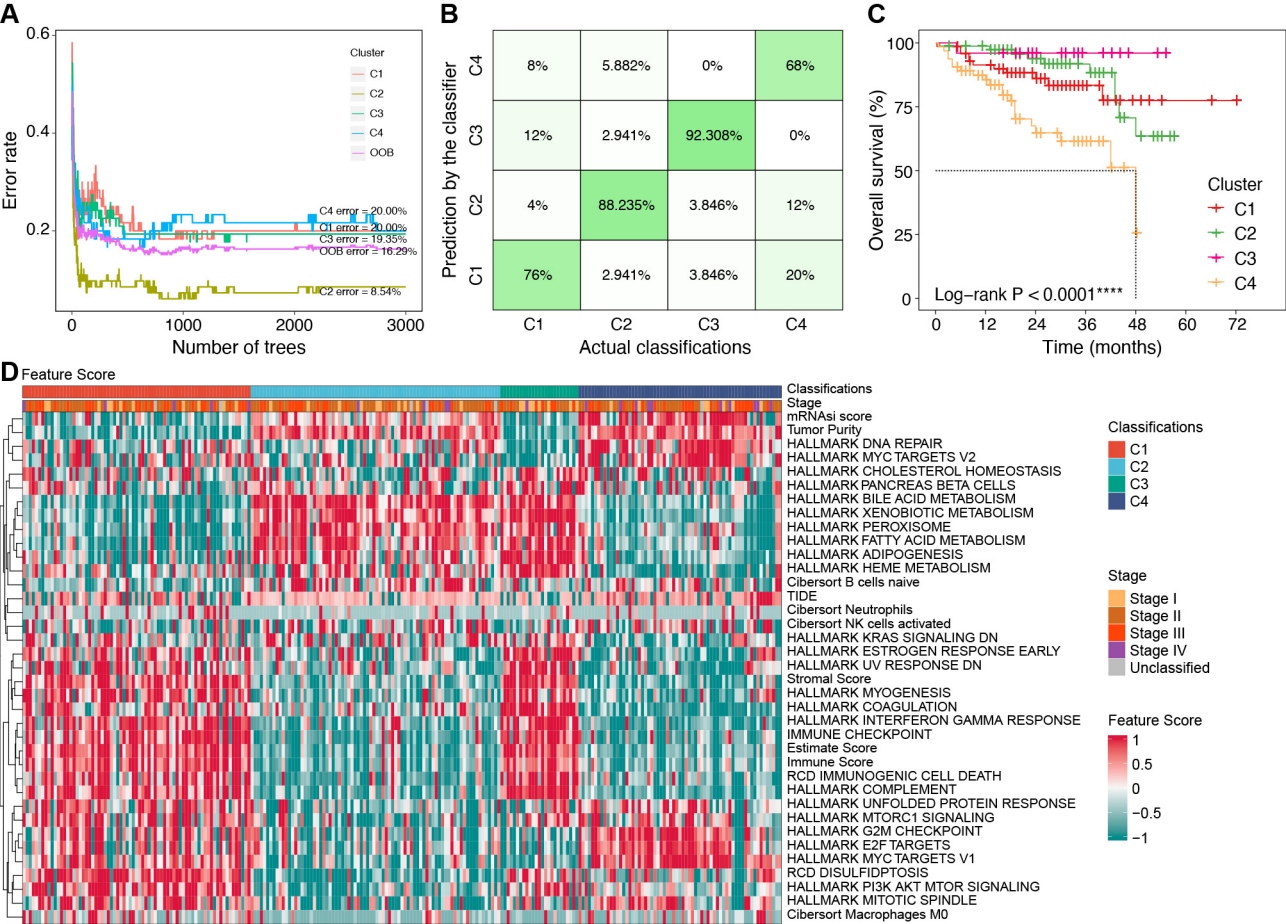
Construction of classifier using the RF algorithm. **(A)** Error rates of the RF model using the 521 cluster-specific marker genes. **(B)** The accuracy in the TCGA internal validation cohort by the RF classifier. **(C)** Survival curves by the RF classifier in the ICGC external validation cohort. **(D)** Characteristics of key feature scores among classifications predicted by the RF classifier in the ICGC external validation cohort. ****P < 0.0001. RF, random forest; TCGA, the Cancer Genome Atlas; ICGC, the International Cancer Genome Consortium.

To efficiently identify the molecular subtypes of patients in real-world settings, we developed a classifier using the random forest (RF) algorithm (**Figure 7A**).

We identified 521 cluster-specific marker genes using the “FindAllMarkers” function (**Supplementary Table S8**). Based on these marker genes, the RF classifier achieved an overall prediction accuracy of 81.82% with a 95% confidence interval (CI) of 73.33%–88.53% in the TCGA internal validation cohort. The prediction accuracy for each subtype by the RF classifier is presented in **Figure 7B**.

In the ICGC external validation cohort, the molecular classifications were predicted using the RF classifier. Compared to the TCGA cohort, the predicted classifications in the ICGC cohort exhibited consistent prognostic characteristics (median OS: C3 > C2 > C1 > C4, *P* < 0.001, **Figure 7C**). We also evaluated the characteristics of FSs (**Figure 7D**), immune cell infiltration (**Supplementary Figure S7A**), and immune genes (**Supplementary Figure S7B**) in the ICGC cohort. These validation results were consistent with the characteristics in the TCGA cohort, which further confirmed the robustness and reliability of our classifier.

Using our classifier, we further tested the hypothesis that C1 is more sensitive to anti-PD1 therapy in two external cohorts including 81 patients. Among them, the PRJEB34724 cohort enrolled 40 patients (6 responders and 34 non-responders), and the GSE202069 cohort enrolled 17 patients (8 responders and 9 non-responders).

**Figure 8:**
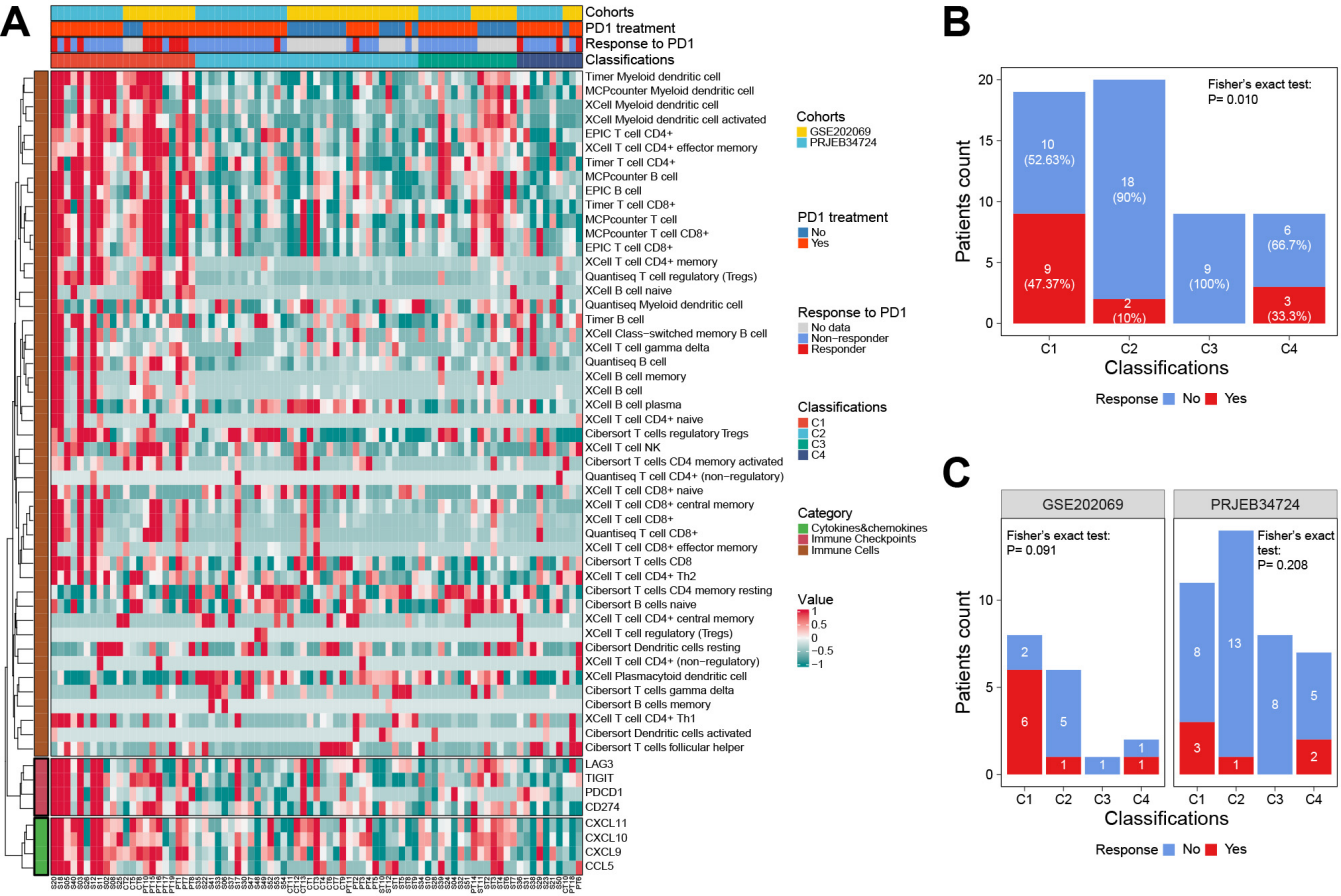
The response to anti-PD1 therapy among classifications in two external validation cohorts. **(A)** The heat map showed the classifications predicted by the RF classifier and the details of response to anti-PD1 therapy. **(B)** The correlation between the response to anti-PD1 therapy and classifications predicted by the RF classifier. **(C)** Subgroup analysis showed the response to anti-PD1 therapy in each cohort. RF, random forest.

The results showed that the immune cell infiltration and ICG expression profiles among the four subtypes were highly similar to the corresponding subtypes in the TCGA cohort (**Figure 8A**). Notably, we confirmed that patients in the C1 subgroup had a higher anti-PD1 response rate of 47.37% (9/19, *P* = 0.01), especially in the GSE202069 cohort with a response rate of 75% (6/8). Moreover, none of the C3-subtype responders were found in either of the two cohorts (**Figures 8B, C**).

### 3.12 Development of the prognostic nomogram

To develop the prognostic nomogram, we identified 70 genes based on the intersection of cluster-specific marker genes and HCCDB prognostic genes. Then seven models were evaluated using the random survival forest (RSF) (**Supplementary Figures S8A–C**), the least absolute shrinkage and selection operator (LASSO) combining 10-fold cross-validation (**Supplementary Figures S8D, E**), and stepwise Cox regression. The details of covariables included in the seven models are shown in **Supplementary Table S9**.

**Figure 9:**
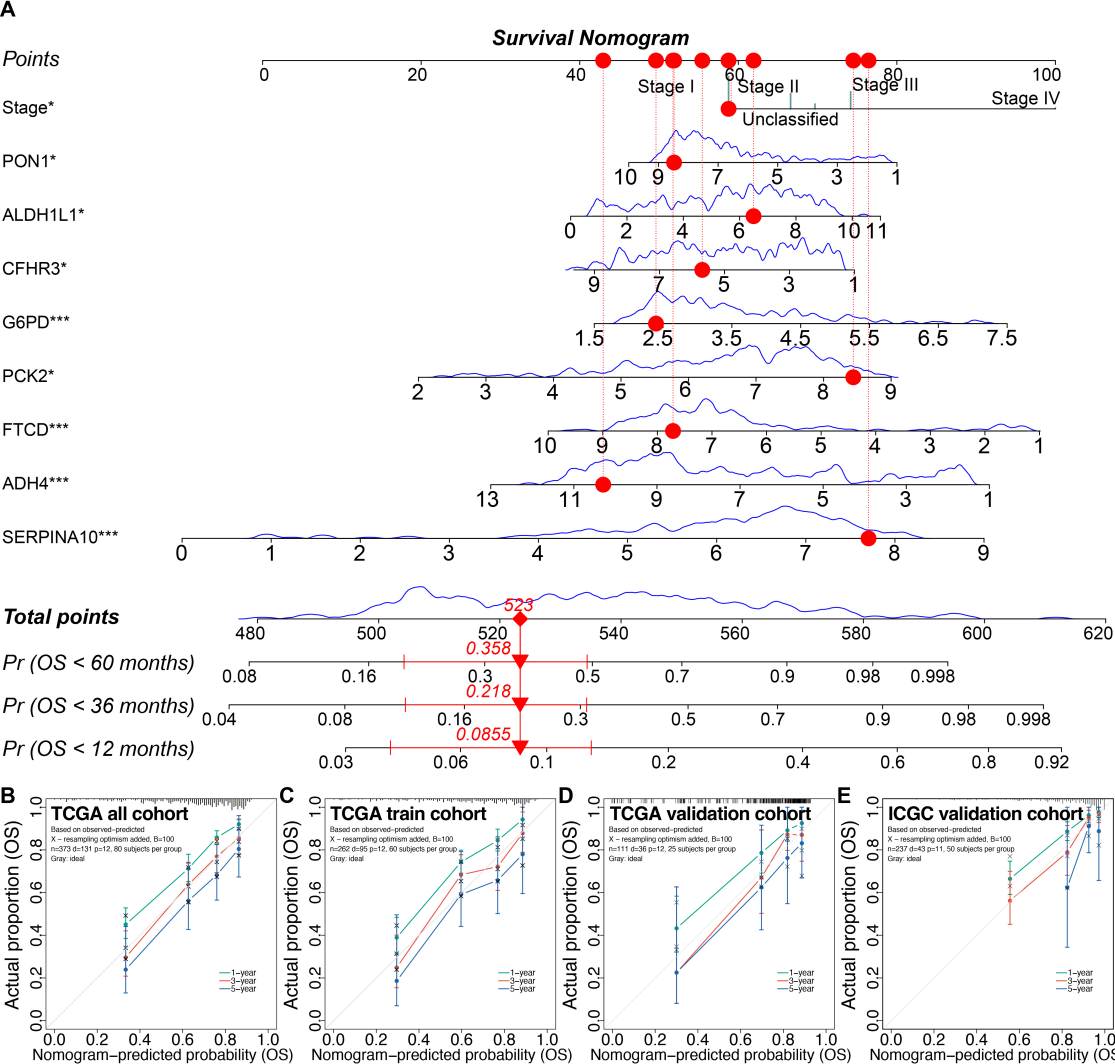
Prognostic nomogram and calibrations in the training and validation cohorts. **(A)** The prognostic nomogram. **(B–E)** The calibration plots of the nomogram in the TCGA all cohort **(B)**, the TCGA training cohort **(C)**, the TCGA inner validation cohort **(D)**, and the ICGC external validation cohort **(E)**. *P < 0.05, ***P < 0.001. TCGA, the Cancer Genome Atlas; ICGC, the International Cancer Genome Consortium.

Using the 3-fold 1000-time-repeation cross-validation, the median concordance index (C-index) of the LASSO combined with stepwise Cox regression and tumor staging (LASSO-Stage) model achieved 0.770 in the training cohort and 0.810 in the TCGA inner validation cohort (**Supplementary Figure S8F**). In addition, we compared the time-dependent receiver operating characteristic curves and found that the LASSO-Stage model had higher values of area under curves at different time points (**Supplementary Figures S8G–J**). Therefore, the 9-variable LASSO-Stage model was relatively simple and accurate, and accordingly was used to develop the prognostic nomogram (**Figure 9A**). The C-index of the nomogram was 0.782 (95% CI, 0.709–0.855) in the external ICGC validation cohort. The calibration curves demonstrated good consistency between the nomogram predictions and actual outcomes (**Figures 9B–E**).

Decision curve analysis indicated that the 9-variable nomogram model was almost as effective as the 26-variable Step-Cox model. Patients could benefit from the nomogram when the risk thresholds range from 0.036 to 0.66 (**Supplementary Figures S9A, B**).

Using the nomogram, the risk scores were calculated for all patients, and the iterative stratification method was used to divide the training cohort into three stratifications (*P* < 0.001). Using the thresholds of the training cohort, patients in the validation cohorts could be divided into significant prognostic stratifications (*P* < 0.01) (**Supplementary Figures S10A–C**). The risk curves and survival status demonstrated that the risk scores of patients predicted by the nomogram were positively related to their death risk (**Supplementary Figures S10D–F**).

To facilitate classification and prognosis prediction, we developed an R package called “oncoClassSurv” (https://github.com/OliveryYL/oncoClassSurv), which has a corresponding desktop executable version (https://github.com/OliveryYL/oncoClassSurv_Expansion/) and a web-based Shiny calculator (https://oncomanager.shinyapps.io/oncoClassSurv/).

### 3.13 Target gene screen and characterization of biological functions

**Figure 10:**
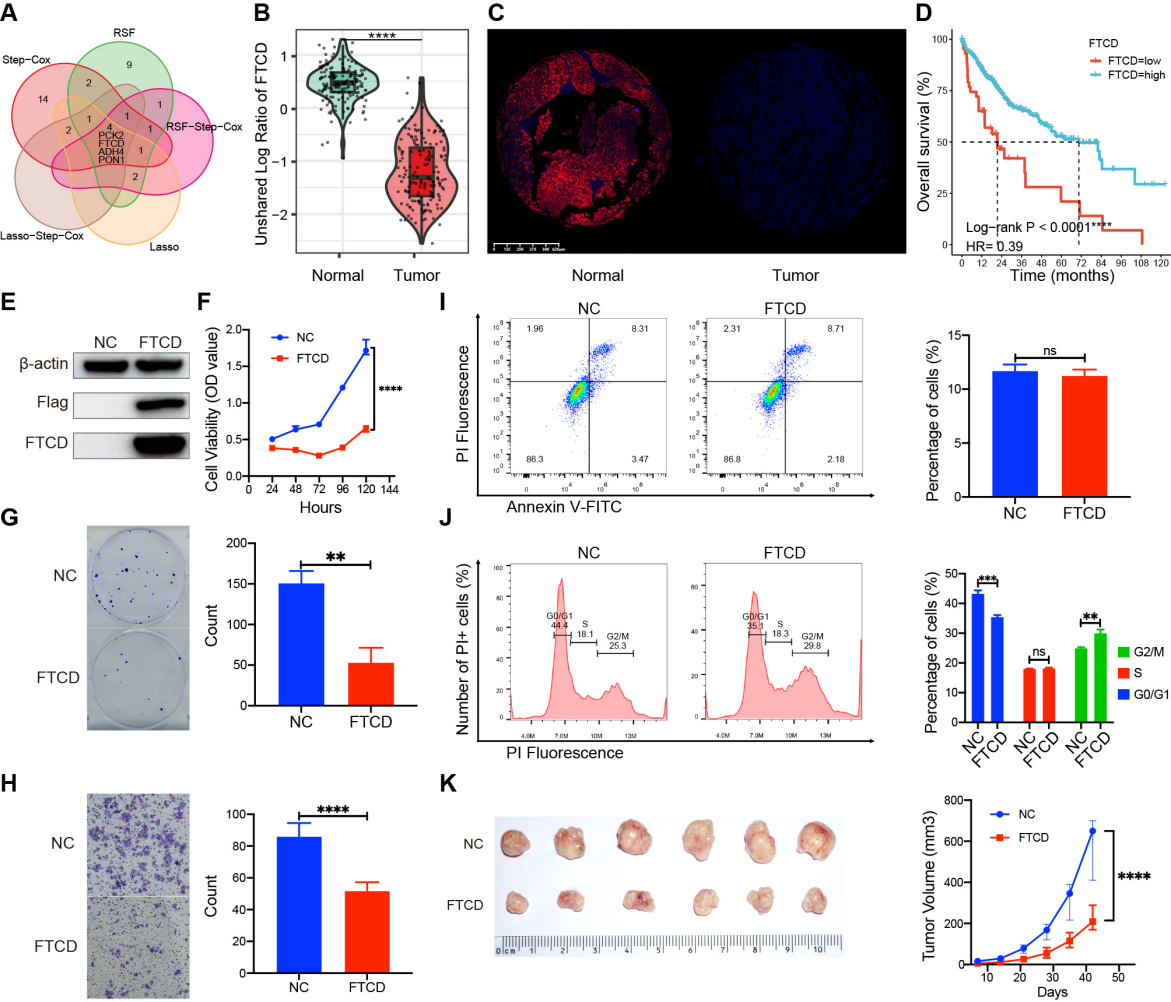
Expression differences, prognostic roles, and biological functions of FTCD. **(A)** Identification of prognostic key genes through Venn analysis. **(B)** The protein expression difference in FTCD was assessed by the CTPAC proteomic database. **(C)** The protein expression difference in FTCD was assessed by the immunofluorescence experiment. **(D)** Survival curves of FTCD expression levels in the TCGA database. **(E)** Protein expression of FTCD-overexpressing Huh7 cell lines by Western blot. **(F)** The Cell Counting Kit-8 (CCK-8) assay assesses the cell viability. **(G)** Colony formation assay assesses the clonogenic ability of cells. **(H)** The transwell assay was used to evaluate the cell migration ability. **(I)** Flow cytometry was used to evaluate changes in cell apoptosis. **(J)** Flow cytometry was used to assess alterations in the cell cycle. **(K)** Subcutaneous tumorigenicity assay in nude mice was used to evaluate the impact on the progression of tumors. **P < 0.01, ***P < 0.001, ****P < 0.0001, ns, not significant.

We identified four crucial prognostic genes through Venn analysis of multiple models (**Figure 10A**). Protein expression profiling and immune fluorescence indicated that FTCD was significantly downregulated in HCC (*P* < 0.001, **Figures 10B, C**). Survival analysis revealed that patients with elevated FTCD expression had a significantly improved prognosis compared to those with diminished expression in the TCGA cohort (*P* < 0.001, **Figure 10D**). FTCD expression was relatively upregulated in C2 and C3 with a favorable prognosis, while it was downregulated in C1 and C4 with a poorer prognosis (**Supplementary Figure S11**).

We established FTCD-overexpressing Huh7 cell lines (Huh7-FTCD) and negative control (Huh7-NC) via lentiviral transduction (**Figure 10E**). Cell proliferation assays indicated that FTCD overexpression significantly inhibited the viability of Huh7 cells (**Figure 10F**). Colony formation assays demonstrated a reduced clonogenic ability in Huh7-FTCD cells (**Figure 10G**); Transwell assays revealed a significantly decreased migration ability in Huh7-FTCD cells (**Figure 10H**). Apoptosis assays showed no significant difference between Huh7-FTCD and Huh7-NC cells (**Figure 10I**), while the Huh7-FTCD cells exhibited G2/M cell cycle arrest compared to Huh7-NC cells (**Figure 10J**). Subcutaneous tumorigenicity assay in nude mice indicated that tumor progression originating from the Huh7-FTCD cells was significantly slower than that from the Huh7-NC cells (**Figure 10K**).

## 4 Discussion

HCC is a highly heterogeneous disease with complex biological characteristics. In the emerging era of precision medicine, it is essential to identify the classifications based on their molecular characteristics. Although several schemes for HCC have been proposed, they often have some limitations. These include a narrow focus on specific dimensions, a lack of validation with external cohorts, and impracticality due to the absence of user-friendly software or overly complex metrics. Thus, the current study was mainly designed to comprehensively characterize HCC heterogeneity across multiple dimensions, develop practical predictive software, and explore the potential therapeutic strategies customized for different subtypes.

Previous studies have proposed several molecular classifications of HCC. A proteogenomic study conducted by Qiang Gao and Jia Fan, et al., categorized hepatitis B virus-associated HCC patients into three distinct subgroups: the metabolic subgroup (S-Mb), the microenvironment dysregulation subgroup (S-Me), and the proliferative subgroup. S-Mb showed enrichment in cancer metabolism and had a favorable prognosis. In contrast, S-Me exhibited enrichment in immunity-related features, with a worse prognosis compared to S-Mb (13). Other classifications mainly focused on a few certain pathways, such as immune or metabolic-related pathways. Chen Yang et al. proposed a three-layered classifier based on the metabolic gene sets (9), while Binghua Li et al. developed a three-layered classifier based on the fatty acid degradation pathway (12); Additionally, Jiao Gong et al. developed a three-layered classifier based on the immunologic and hallmark gene sets (10). Montironi et al. described the immune genome background of HCC, dividing it into inflammatory and non-inflammatory tumors (11). Recently, the study by Shimada S et al. summarized the past development of molecular classifications of HCC, highlighting the importance of characterizing HCC subtypes (34). Our study contributes to this field by developing a molecular typing system with four subtypes based on proliferation, microenvironment (immune/stromal), and metabolic activity. We compared our subtypes with previously identified subtypes and found interesting overlaps. For instance, our C1 subtype shares similarities with previously reported subtypes like MFP-IE, inflammatory, or FAD-F1 subtype, while C2, with its CTNNB1 mutations and immune therapy response patterns, is consistent with previous findings. This comparison helps explain our classification system and provides insights into HCC heterogeneity.

We appreciate their groundbreaking studies. However, these schemes also have acknowledged limitations, as mentioned previously. To characterize the complex heterogeneity of HCC, we conducted comprehensive analyses across multiple FSs dimensions. In addition, we integrated single-cell data analysis and combined the TIME-MFP typing system to scrutinize the characteristics of subtypes. Among the initial 89 FSs, the selected signal pathways act as significant roles across various malignancies (35–40); mRNAsi can represent the stemness characteristics closely related to tumor proliferation and progression (41, 42); Metrics such as TIDE score, MFP typing, immune score, immune checkpoint, and immune cell infiltration can reflect the TIME and contribute to clarifying the relationship between subtypes and immune response (25–27, 30). The integrated tumor metabolic score can reflect the metabolic reprogramming characteristics of HCC, and the m6A and m5C gene sets are hot fields involving RNA modification (43–45). The 37 key FSs were rigorously selected from the initial FSs through differential expression and survival analyses to ensure their relevance to HCC’s biological characteristics. Therefore, our classification system encompasses multiple critical dimensions, providing robust evidence for precise management and research.

In this study, we performed unsupervised clustering analysis on the FSs derived from TCGA-LIHC and generated a clustering heatmap of the FSs (**Figure 4E**). After predicting molecular classifications on the ICGC-LIRI-JP dataset using our tool, we generated another clustering heatmap (**Figure 7D**). According to the FSs clustering heatmaps, HCC exhibited complex heterogeneity and could be stratified into four subtypes (C1–C4) from the three major fields: stemness proliferation, metabolism, and microenvironment (stroma-riched or immunity-riched). Stemness-proliferation-related FSs include G2M, MYC, E2F, PI3K AKT mTOR, mRNAsi, and DNA repair activity, etc. From the perspective of stemness proliferation, both C1 and C4 exhibited high G2M, MYC, E2F activities, and MKI67 expression levels. Besides, C1 had high PI3K AKT mTOR activity, while C4 had high mRNAsi and DNA repair activity. Therefore, both C1 and C4 exhibited high stemness-proliferation characteristics. In contrast, C2 and C3 had lower levels of MKI67, mRNAsi, G2M, MYC, and E2F, indicating a low proliferative phenotype. Metabolism-related FSs include HALLMARK BILE ACID METABOLISM, HALLMARK XENOBIOTIC METABOLISM, HALLMARK ADIPOGENESIS, and HALLMARK FATTY ACID METABOLISM, etc. From the perspective of metabolism, C1 and C4 were hypometabolic, while C2 and C3 were hypermetabolic. Microenvironment-related FSs can be divided into two subcategories: immune-related FSs and stroma-related FSs. Immune-related FSs include immune cell infiltration proportions, immune score, TIDE, RCD immunogenic cell death, HALLMARK COMPLEMENT, immune checkpoint, and HALLMARK INTERFERON GAMMA RESPONSE. Stroma-related FSs include stromal score, estimate score, and tumor purity. From the perspective of microenvironment, both C1 and C3 exhibited rich microenvironment phenotypes. C1 was particularly immune-rich with high lymphocyte infiltration and overexpression of multiple HLA genes and suppressive immune checkpoint genes. Most of C1 belonged to the MFP-IE subtype (37.50%) with a high potential for response to anti-PD1 therapy (30); C3 was mainly a stroma-rich subtype, rich in CAF, with overexpression of HLA genes, and diminished expression of suppressive immune checkpoint genes. Most patients of C3 were MFP-F subtype (47.67%) and MFP-IE subtype (32.56%). C2 and C4 were stroma-desert, immune-desert, and highly tumor-purity subtypes, with most of C2 (64.29%) and C4 (72.15%) belonging to the MFP-D subtype. Thus, considering the correlation with immune and MFP classifications, it can be inferred that C1 may be more suitable for anti-PD1 treatment.

Considering the characteristics of pseudo-temporal trajectory, staging, and prognosis, C3 was the closest subtype to normal tissue and in the early phase of the disease, with the most favorable prognosis. C2 was in the middle phase of the disease with a good prognosis, only inferior to C3. C1 was in the middle or late phase of the disease with a poor prognosis. C4 was in the late phase of the disease with the worst prognosis.

Gene mutations are known to drive various tumors (46). Multiple studies have indicated that TP53 mutation is an important tumor driver associated with poor prognosis (47–49). In the current study, the mutation frequency of TP53 (C4 > C1 > C2 > C3) was closely correlated with the prognosis of different subtypes (median OS: C4 < C1 < C2 < C3). Previous studies suggested that patients with wild-type TP53 and high expression of MDM2 would be more sensitive to MDM2 inhibitors (50). Interestingly, most C3 patients fit this condition, which accounted for their high sensitivity to nutlin-3a (**Supplementary Figure S5**); In addition, the mutation rates of CTNNB1 were higher in C2, while they were both lower in C1 and C3. CTNNB1 encodes β-catenin, and the previous study suggested its mutations can activate the WNT/β-catenin signaling pathway, which is commonly found in immunotherapy-unresponsive individuals (32). Accordingly, we made the following inference that C2 is relatively not suitable for anti-PD1 therapy due to their immune-desert characteristics and higher CTNNB1 mutations. This inference can be verified by external cohorts experiencing anti-PD1 therapy in the following analysis. Moreover, we identified the most sensitive drugs for each subtype, which could provide references for further research.

We further investigated the cluster characteristics at the single-cell level. The results of the single-cell analysis exhibited high consistency among clusters with the immune-related findings from the bulk RNA-Seq data in the TCGA cohort. For instance, C1 is enriched in immune cells, while C3 is enriched in CAF. C2 is predominantly composed of malignant cells with a lack of immune cells. These findings provide insights into the varied responses to anti-PD1 therapy among clusters from the single-cell perspective.

To verify our inference of sensitivity to immune therapy in external cohorts, we first needed to accurately predict their classifications. To achieve this, we constructed a four-layer classifier based on the TCGA cohort using the RF algorithm, which performed well in the external ICGC cohort. The predicted classifications of the external ICGC cohort showed consistent characteristics, including prognostic outcomes and FSs among distinct classifications, compared to the TCGA cohort. Using the robust classifier, we further conducted another external validation in two datasets experiencing anti-PD1 therapies to evaluate the response to anti-PD1 therapy. The encouraging results confirmed our inference that the C1 subtype has the highest response to anti-PD1 therapy.

We conducted multivariate analyses to assess the prognostic values of the selected genes using machine learning algorithms. The critical classification-specific prognostic genes were selected and used to develop a prognostic nomogram. This nomogram has excellent prediction ability and can provide personalized prognostic evaluations.

Based on the above investigations, we further developed practical prediction software that can be applied to evaluate the classifications and prognosis of HCC, including three versions: local R program, desktop executable software, and web-based application. These tools can also utilize customized expression profiles and classification-specific marker genes to expand to other diseases.

Furthermore, we propose that the functional characterization for molecular typing marker genes can serve as a novel anti-tumor target screening approach. This screening strategy holds promise for precise patient screening and decision-making based on molecular classifications in the future. To explore targets with high potential therapeutic values, we performed Venn analysis and expression profiling differential analysis for the prognostic genes, and identified FTCD as a critical prognostic gene with significant expression differences.

In the clinical cohort, FTCD expression is downregulated in C1 and C4 subtypes, which is correlated with increased tumor malignancy and poorer patient outcomes. In contrast, C2 and C3 subtypes exhibit FTCD overexpression with more favorable prognoses.

A previous study indicated that hepatocyte-specific knockout of FTCD can promote chronic diethylnitrosamine-induced and spontaneous HCC in mice. The Loss of FTCD upregulated peroxisome proliferator-activated receptor (PPAR)γ and sterol regulatory element-binding protein 2 (SREBP2) by regulating the PTEN/Akt/mTOR signaling axis, leading to lipid accumulation and hepatocarcinogenesis (51).

Our investigation into FTCD’s biological roles further revealed its tumor-suppressive function. A series of cell and animal experiments suggested that FTCD overexpression can significantly inhibit the progression both *in vitro* and *in vivo* (**Figure 10**). Thus, diminished FTCD expression is a key driver of disease progression in C1 and C4 subtypes. Enhancing FTCD expression in these patients may lead to substantial therapeutic gains. In future scientific research and clinical management, we may refine tumor biological behavior and patient outcomes through targeted delivery methods, such as mRNA-liposome nanomedicine, to boost FTCD expression levels in HCC tissues. Accordingly, the FTCD gene emerges as a viable therapeutic target for C1 and C4 patients. Certainly, these findings warrant further investigation for validation.

## 5 Conclusions

In summary, we proposed a novel molecular classification scheme for HCC based on the multidimensional FSs. The four-layer classification scheme reveals the complex heterogeneity of HCC through comprehensive characterization. We also developed and validated predictive software. Notably, our research confirmed that the C1 subtype is more responsive to anti-PD1 treatment, and identified FTCD as a promising therapeutic target, particularly for C1 and C4. These findings provide robust evidence for individualized disease evaluation, decision-making, and further scientific research.

## Data Availability

The original contributions presented in the study are included in the article/**Supplementary Material**. Further inquiries can be directed to the corresponding authors.

## References

[1] Sung H Ferlay J Siegel RL Laversanne M Soerjomataram I Jemal A. Global Cancer Statistics 2020: GLOBOCAN estimates of incidence and mortality worldwide for 36 cancers in 185 countries. CA Cancer J Clin. (2021) 71:209–49. doi: 10.3322/caac.21660 33538338

[2] Singal AG Lampertico P Nahon P. Epidemiology and surveillance for hepatocellular carcinoma: New trends. J Hepatol. (2020) 72:250–61. doi: 10.1016/j.jhep.2019.08.025 PMC6986771 31954490

[3] Dhanasekaran R Suzuki H Lemaitre L Kubota N Hoshida Y. Molecular and immune landscape of hepatocellular carcinoma to guide therapeutic decision making. Hepatology. [Preprint]. (2023). doi: 10.1097/hep.0000000000000513 PMC10713867 37300379

[4] Zhang W He H Zang M Wu Q Zhao H Lu LL. Genetic features of aflatoxin-associated hepatocellular carcinoma. Gastroenterology. (2017) 153:249–62.e2. doi: 10.1053/j.gastro.2017.03.024 28363643

[5] Wege H Li J Ittrich H. Treatment lines in hepatocellular carcinoma. Visc Med. (2019) 35:266–72. doi: 10.1159/000501749 PMC6738173 31602390

[6] Suresh D Srinivas AN Prashant A Harikumar KB Kumar DP. Therapeutic options in hepatocellular carcinoma: a comprehensive review. Clin Exp Med. (2023) 23:1901–16. doi: 10.1007/s10238-023-01014-3 36780119

[7] Nakagawa S Wei L Song WM Higashi T Ghoshal S Kim RS. Molecular liver cancer prevention in cirrhosis by organ transcriptome analysis and lysophosphatidic acid pathway inhibition. Cancer Cell. (2016) 30:879–90. doi: 10.1016/j.ccell.2016.11.004 PMC5161110 27960085

[8] Goossens N Sun X Hoshida Y. Molecular classification of hepatocellular carcinoma: potential therapeutic implications. Hepat Oncol. (2015) 2:371–9. doi: 10.2217/hep.15.26 PMC4662420 26617981

[9] Yang C Huang X Liu Z Qin W Wang C. Metabolism-associated molecular classification of hepatocellular carcinoma. Mol Oncol. (2020) 14:896–913. doi: 10.1002/1878-0261.12639 31955511 PMC7138397

[10] Gong J Li R Chen Y Zhuo Z Chen S Cao J. HCC subtypes based on the activity changes of immunologic and hallmark gene sets in tumor and nontumor tissues. Brief Bioinform. (2021) 22:1–13. doi: 10.1093/bib/bbaa427 33515024

[11] Montironi C Castet F Haber PK Pinyol R Torres-Martin M Torrens L. Inflamed and non-inflamed classes of HCC: a revised immunogenomic classification. Gut. (2023) 72:129–40. doi: 10.1136/gutjnl-2021-325918 PMC9395551 35197323

[12] Li B Li Y Zhou H Xu Y Cao Y Cheng C. Multiomics identifies metabolic subtypes based on fatty acid degradation allocating personalized treatment in hepatocellular carcinoma. Hepatology. (2024) 79:289–306. doi: 10.1097/HEP.0000000000000553 37540187 PMC10789383

[13] Gao Q Zhu H Dong L Shi W Chen R Song Z. Integrated proteogenomic characterization of HBV-related hepatocellular carcinoma. Cell. (2019) 179:561–77. doi: 10.1016/j.cell.2019.08.052 31585088

[14] Hou Y Pang H Xu X Zhao D. Identifying and validating an angiogenesis-related signature for the prognosis of head and neck squamous cell carcinoma. Curr Medicinal Chem. (2024) 31:1–15. doi: 10.2174/0109298673306245240514064119 38752633

[15] Huang Y Yin D Wu L. Identification of cuproptosis-related subtypes and development of a prognostic signature in colorectal cancer. Sci Rep. (2022) 12:1–10. doi: 10.1038/s41598-022-22300-2 36253436 PMC9576756

[16] Qi L Xu R Ren X Zhang W Yang Z Tu C. Comprehensive profiling reveals prognostic and immunogenic characteristics of necroptosis in soft tissue sarcomas. Front Immunol. (2022) 13:877815. doi: 10.3389/fimmu.2022.877815 35663937 PMC9159500

[17] Ren J Yang J Na S Wang Y Zhang L Wang J. Comprehensive characterisation of immunogenic cell death in melanoma revealing the association with prognosis and tumor immune microenvironment. Front Immunol. (2022) 13:998653. doi: 10.3389/fimmu.2022.998653 36211436 PMC9538190

[18] Diao X Guo C Li S. Identification of a novel anoikis-related gene signature to predict prognosis and tumor microenvironment in lung adenocarcinoma. Thorac Cancer. (2023) 14:320–30. doi: 10.1111/1759-7714.14766 PMC9870742 36507553

[19] Liu X Nie L Zhang Y Yan Y Wang C Colic M. Actin cytoskeleton vulnerability to disulfide stress mediates disulfidptosis. Nat Cell Biol. (2023) 25:404–14. doi: 10.1038/s41556-023-01091-2 PMC10027392 36747082

[20] Wang T Guo K Zhang D Wang H Yin J Cui H. Disulfidptosis classification of hepatocellular carcinoma reveals correlation with clinical prognosis and immune profile. Int Immunopharmacol. (2023) 120:1–15. doi: 10.1016/j.intimp.2023.110368 37247499

[21] Zhang J Wang Z Zhang X Dai Z Zhi-Peng W Yu J. Large-scale single-cell and bulk sequencing analyses reveal the prognostic value and immune aspects of CD147 in pan-cancer. Front Immunol. (2022) 13:810471. doi: 10.3389/fimmu.2022.810471 35464411 PMC9019465

[22] Malta TM Sokolov A Gentles AJ Burzykowski T Poisson L Weinstein JN. Machine learning identifies stemness features associated with oncogenic dedifferentiation. Cell. (2018) 173:338–54. doi: 10.1016/j.cell.2018.03.034 PMC5902191 29625051

[23] Chen W Liao Y Sun P Tu J Zou Y Fang J. Construction of an ER stress-related prognostic signature for predicting prognosis and screening the effective anti-tumor drug in osteosarcoma. J Trans Med. (2024) 22:66. doi: 10.1186/s12967-023-04794-0 PMC10792867 38229155

[24] Hu F-F Liu C-J Liu L-L Zhang Q Guo A-Y. Expression profile of immune checkpoint genes and their roles in predicting immunotherapy response. Brief Bioinform. (2020) 22:1–12. doi: 10.1093/bib/bbaa176 32814346

[25] Jiang P Gu S Pan D Fu J Sahu A Hu X. Signatures of T cell dysfunction and exclusion predict cancer immunotherapy response. Nat Med. (2018) 24:1550–8. doi: 10.1038/s41591-018-0136-1 PMC6487502 30127393

[26] Fu J Li K Zhang W Wan C Zhang J Jiang P. Large-scale public data reuse to model immunotherapy response and resistance. Genome Med. (2020) 12:1–8. doi: 10.1186/s13073-020-0721-z PMC7045518 32102694

[27] Zhao Y Yu J Zheng C Zhou B. Establishment of a prognostic model for hypoxia-associated genes in OPSCC and revelation of intercellular crosstalk. Front Immunol. (2024) 15:1371365. doi: 10.3389/fimmu.2024.1371365 38887298 PMC11181350

[28] Hänzelmann S Castelo R Fau - Guinney J Guinney J. GSVA: gene set variation analysis for microarray and RNA-seq data. BMC Bioinf. (2013) 14:1–15. doi: 10.1186/1471-2105-14-7 PMC3618321 23323831

[29] Wilkerson MD Hayes DN. ConsensusClusterPlus: a class discovery tool with confidence assessments and item tracking. Bioinformatics. (2010) 26:1572–3. doi: 10.1093/bioinformatics/btq170 PMC2881355 20427518

[30] Bagaev A Kotlov N Nomie K Svekolkin V Gafurov A Isaeva O. Conserved pan-cancer microenvironment subtypes predict response to immunotherapy. Cancer Cell. (2021) 39:845–65. doi: 10.1016/j.ccell.2021.04.014 34019806

[31] Hao Y Hao S Andersen-Nissen E Mauck WM III Zheng S Butler A. Integrated analysis of multimodal single-cell data. Cell. (2021) 184:3573–87. doi: 10.1016/j.cell.2021.04.048 PMC8238499 34062119

[32] Hong JY Cho HJ Sa JK Liu X Ha SY Lee T. Hepatocellular carcinoma patients with high circulating cytotoxic T cells and intra-tumoral immune signature benefit from pembrolizumab: results from a single-arm phase 2 trial. Genome Med. (2022) 14:1–15. doi: 10.1186/s13073-021-00995-8 34986867 PMC8734300

[33] Li B Cao Y Li Y Cheng C Yu D. Letter to the editor: the inflamed subclass predicts immunotherapy response – external validations. Gut. (2023) 72:1224. doi: 10.1136/gutjnl-2022-328130 35842234

[34] Shimada S Tanaka S. The chronicles of hepatocellular carcinoma classification: Subtyping, modeling, and treatment. Hepatology. (2024) 79:261–3. doi: 10.1097/HEP.0000000000000588 37651225

[35] Li X Lei J Shi Y Peng Z Gong M Shu X. Developing a riskscore model based on angiogenesis-related lncRNAs for colon adenocarcinoma prognostic prediction. Curr Medicinal Chem. (2024) 31:2449–66. doi: 10.2174/0109298673277243231108071620 37961859

[36] Wang W Guo H Wu S Xian S Zhang W Zhang R. Construction of metastasis-specific regulation network in ovarian cancer based on prognostic stemness-related signatures. Reprod Sci. (2023) 30:2634–54. doi: 10.1007/s43032-022-01134-3 36940084

[37] Xiao J Zheng L Liu J. Comprehensive analysis of the aberrance and functional significance of ferroptosis in gastric cancer. Front Pharmacol. (2022) 13. doi: 10.3389/fphar.2022.919490 PMC9315307 35903347

[38] Xue Y Jiang X Wang J Zong Y Yuan Z Miao S. Effect of regulatory cell death on the occurrence and development of head and neck squamous cell carcinoma. biomark Res. (2023) 11:2. doi: 10.1186/s40364-022-00433-w 36600313 PMC9814270

[39] Liu R-J Yu X-D Yan S-S Guo Z-W Zao X-B Zhang Y-S. Ferroptosis, pyroptosis and necroptosis in hepatocellular carcinoma immunotherapy: Mechanisms and immunologic landscape (Review). Int J Oncol. (2024) 64:63. doi: 10.3892/ijo.2024.5651 38757345 PMC11095606

[40] Zhou Q Gao X Xu H Lu X. Non-apoptotic regulatory cell death scoring system to predict the clinical outcome and drug choices in breast cancer. Heliyon. (2024) 10:e31342. doi: 10.1016/j.heliyon.2024.e31342 38813233 PMC11133894

[41] Fu S Tan Z Shi H Chen J Zhang Y Guo C. Development of a stemness-related prognostic index to provide therapeutic strategies for bladder cancer. NPJ Precis Oncol. (2024) 8:14. doi: 10.1038/s41698-024-00510-3 38245587 PMC10799910

[42] Weng M Li T Zhao J Guo M Zhao W Gu W. mRNAsi-related metabolic risk score model identifies poor prognosis, immunoevasive contexture, and low chemotherapy response in colorectal cancer patients through machine learning. Front Immunol. (2022) 13:950782. doi: 10.3389/fimmu.2022.950782 36081499 PMC9445443

[43] Xu Q Xu H Deng R Li N Mu R Qi Z. Landscape of prognostic m6A RNA methylation regulators in hepatocellular carcinoma to aid immunotherapy. Front Cell Dev Biol. (2021) 9:669145. doi: 10.3389/fcell.2021.669145 34422799 PMC8375309

[44] Lv Z Ran R Yang Y Xiang M Su H Huang J. The interplay between N6-methyladenosine and precancerous liver disease: molecular functions and mechanisms. Discovery Oncol. (2023) 14:1–18. doi: 10.1007/s12672-023-00695-2 PMC10212880 37227534

[45] Huang Z Pan J Wang H Du X Xu Y Wang Z. Prognostic significance and tumor immune microenvironment heterogenicity of m5C RNA methylation regulators in triple-negative breast cancer. Front Cell Dev Biol. (2021) 9:657547. doi: 10.3389/fcell.2021.657547 33928086 PMC8076743

[46] Martínez-Jiménez F Muiños F Sentís I Deu-Pons J Reyes-Salazar I Arnedo-Pac C. A compendium of mutational cancer driver genes. Nat Rev Cancer. (2020) 20:555–72. doi: 10.1038/s41568-020-0290-x 32778778

[47] Yang C Huang X Li Y Chen J Lv Y Dai S. Prognosis and personalized treatment prediction in TP53-mutant hepatocellular carcinoma: an in silico strategy towards precision oncology. Brief Bioinform. (2021) 22:1–13. doi: 10.1093/bib/bbaa164 32789496

[48] Shi C Liu S Tian X Wang X Gao P. A TP53 mutation model for the prediction of prognosis and therapeutic responses in head and neck squamous cell carcinoma. BMC Cancer. (2021) 21:1–13. doi: 10.1186/s12885-021-08765-w 34530752 PMC8447564

[49] Ye S Zhao X-Y Hu X-G Li T Xu Q-R Yang H-M. TP53 and RET may serve as biomarkers of prognostic evaluation and targeted therapy in hepatocellular carcinoma. Oncol Rep. (2017) 37:2215–26. doi: 10.3892/or.2017.5494 PMC5367355 28350084

[50] Wang S Chen F-E. Small-molecule MDM2 inhibitors in clinical trials for cancer therapy. Eur J Med Chem. (2022) 236:1–20. doi: 10.1016/j.ejmech.2022.114334 35429910

[51] Wang S Zhou Y Yu R Ling J Li B Yang C. Loss of hepatic FTCD promotes lipid accumulation and hepatocarcinogenesis by upregulating PPARγ and SREBP2. JHEP Rep. (2023) 5:100843. doi: 10.1016/j.jhepr.2023.100843 37675273 PMC10477690

