# Supplementary material for: A novel molecular classification system based on the molecular feature score identifies patients sensitive to immune therapy and target therapy
Source: Front Immunol. 2024 Nov 26;15:1466069. doi: 10.3389/fimmu.2024.1466069 (PMC11628386; doi:10.3389/fimmu.2024.1466069)
Supplement: Supplementary file 2 [file DataSheet1.pdf]

Supplementary Materials

This file includes:

1. Supplementary Materials and Methods.
2. Supplementary References
3. Supplementary Figures

Supplementary Materials and Methods

1.1 Data collection

The "TCGAbiolinks" package was used to download the following data from the Cancer Genome Atlas (TCGA) program: bulk RNA sequencing (RNA-seq) transcriptome, single nucleotide variation (SNV), and clinical information. In the development of the classifier and nomogram, 70% of samples from the TCGA-LIHC dataset were randomly sampled for training, while the other 30% were used for internal validation.

The bulk RNA-seq transcriptome data and clinical information from the International Cancer Genome Consortium (ICGC) were downloaded from https://dcc.icgc.org/releases/current/Projects/LIRI-JP. In the development of the classifier and nomogram, the ICGC-LIRI-JP dataset was used for external validations.

The scRNA-Seq dataset (GSE151530) was downloaded from Gene Expression Omnibus (GEO) (https://www.ncbi.nlm.nih.gov/geo/query/acc.cgi?acc=GSE151530), which contains 32 samples from 25 patients with hepatocellular carcinoma (HCC) (1). GSE151530 was mainly used for the characterization of clusters by combining with the bulk RNA-Seq data.

To externally validate response to anti-PD1 therapy among subtypes predicted by the classifier, the GSE202069 dataset was downloaded from GEO (https://www.ncbi.nlm.nih.gov/geo/query/acc.cgi?acc=GSE202069), and the PRJEB34724 dataset was downloaded from the European Nucleotide Archive (ENA) (https://www.ebi.ac.uk/ena/browser/view/PRJEB34724?show=xrefs).

To acquire the consistent genes with significant differential expression and prognostic values, data was downloaded from the HCCDB database (http://lifeome.net/database/hccdb/home.html) which contains 15 public HCC datasets (2).

Furthermore, to investigate the correlation between the clusters and tumor immune microenvironment (TIME), we combined another classification criterion, called molecular functional portrait (MFP). Tumor samples were classified into four MFP subtypes: immune-enriched, fibrotic (IE/F), immune-enriched (IE), fibrotic (F), and depleted (D). Patients with the MFP-IE subtype were considered sensitive to immune checkpoint inhibitors (3).

Data process and analysis were mainly performed by R version 4.3.1 (https://cran.r-project.org/).

1.2 Calculation of feature scores (FSs)

1.2.1 Gene set variation analysis (GSVA) scoring of gene sets (4)

The GSVA scoring was conducted using the following 61 gene sets: 50 hallmark gene sets from the Molecular Signatures Database (MSigDB, https://www.gsea-msigdb.org/gsea/msigdb) and another 11 crucial gene sets (5). Specifically, the 11 gene sets consist of 1 merged metabolic gene set integrated from 34 metabolism gene sets annotated as "KEGG" from the MSigDB, 1 immune check point gene set (6), 7 regulated cell death (RCD) related gene sets (including RCD_FERROPTOSIS (MSigDB), RCD_CUPROPTOSIS (7), RCD_NECROPTOSIS (8), RCD_PYROPTOSIS (MSigDB), RCD_IMMUNOGENIC_CELL_DEATH (9), RCD_ANOIKIS (10), RCD_DISULFIDPTOSIS (11, 12)), 1 m5C gene set (13), and 1 m6A gene set (14, 15). After the normalization of the expression data by the "limma" package (16), these gene sets were used to calculate the feature scores (FSs) for every sample from the TCGA and ICGC cohorts by GSVA scoring.

1.2.2 Curated scores

We selected six curated scores, including mRNAsi, TIDE, estimate score, immune score, stromal score, and tumor purity, among which the latter four scores were calculated by the R package of "estimate" (17). The calculation of mRNAsi score was based on the study of Malta et al (18), while the TIDE score was based on the studies of Jiang and Fu et al (19, 20).

1.2.3 Immune cell infiltration abundance

A total of six different immune infiltration algorithms (Cibersort, Quantiseq, Timer, MCPcounter, XCell, and EPIC) were used to estimate the abundance of tissue-infiltrating immune cells by the R package of "immunedeconv" (https://github.com/omnideconv/immunedeconv) (21).

1.3 Screening of significant FSs and the proposal of a novel molecular classification scheme

1.3.1 Differential analysis of FSs

A total of 89 FSs were enrolled in the current study: 61 GSVA scores of gene sets, 6 curated scores, and 22 immune cell infiltration proportions by the Cibersort algorithm.

Then the "limma" package was used to evaluate the differential significance of FSs between the normal and tumor tissues in the TCGA and ICGC cohorts, respectively. FSs with adjusted $P < 0.05$ and $|\log2(\text{FoldChange})| > 0$ were considered significant.

1.3.2 Survival analysis of FSs

FSs were classified as relatively high or low level using the "surv_cutpoint" function from "survminer" package (https://rdocumentation.org/packages/survminer/). Subsequently, the Kaplan-Meier method and log-rank test were used to compare the difference of overall survival (OS) between the high and low levels. The univariate Cox regression was performed in the TCGA and ICGC cohorts, respectively.

1.3.3 Screening of significant FSs by Venn analysis

To identify the consistently significant FSs, Venn analysis was performed based on the results of differential analysis and Kaplan-Meier survival analysis on FSs.

1.3.4 Unsupervised clustering analysis

The consistently significant FSs by the Venn analysis were enrolled in the unsupervised clustering analysis using the “ConsensusClusterPlus” package. The appropriate number of clusters was determined according to the consensus cumulative distribution function (CDF) plot and the delta area plot (22).

1.4 Identification of novel molecular classifications

1.4.1 Identification of the cluster-specific marker genes

The function of “FindAllMarkers” from the “Seurat” package was used to calculate the foldchange and *P* value of each gene among clusters (23). Genes with $P < 0.05$ and |log2(FoldChange)| > 0.5 were considered as the cluster-specific marker genes.

1.4.2 Enrichment analysis

Gene set enrichment analysis (GSEA) was conducted by the “GSEABase” package (24). Gene sets from the MSigDB database were used as the reference gene sets. Subsequently, we selected the Hallmark signaling pathways and KEGG signaling pathways from the Msigdb database for visualization. Compared with other subtypes as a whole, the log2FoldChange was calculated for each subtype separately, and then GSEA was performed on each subtype.

1.4.3 Pseudo-temporal trajectory analysis

The pseudo-temporal trajectory analysis was performed using the “monocle” package (25). Specifically, the dimensionality reduction analysis was conducted with the “reduceDimension” function. The discriminative dimensionality reduction with trees (DDRTree) was used as the dimension reduction method. Subsequently, the pseudo-temporal trajectory plots were visualized with the function of plot_cell_trajectory.

1.4.4 SNV analysis

Using the “GenVisR” package, we visualize the SNV profile of the top 20 genes with the highest mutations in the TCGA cohort (26).

1.4.5 Drug sensitivity analysis

The “oncoPredict” package was used to train drug sensitivity models on the Genomics of Drug Sensitivity in Cancer (GDSC) v2 dataset, and then the drug sensitivity of all samples was predicted (27). Drugs with log2(FoldChange) < -0.5 and $P < 0.05$ were considered significantly sensitive, while drugs with log2(FoldChange) > 0.5 and $P < 0.05$ were considered significantly resistant.

1.4.6 Identification of single-cell level

HCC samples from the GSE151530 dataset were extracted for our analyses. The “Seurat” package was applied to the scRNA-Seq data for filtering genes and cells, normalization, PCA, and t-distributed stochastic neighbor embedding (t-SNE) (23). The quality control criteria apply to the uploader (28). Cells with > 700 detected genes, < 20% of mitochondrial gene expression, < 0.1% of hemoglobin genes, and > 3% of ribosomal genes were reserved. Genes detected in < 3 cells were omitted. Doublets were removed from the Seurat object using the “DoubletFinder” package (29). The data of high-quality cells were normalized after filtering and highly variable genes were identified for subsequent analyses. Significant principal components (PCs) were determined by performing PCA on these genes. The top 20 PCs were used for clustering the cells. The marker genes of each cluster were identified by performing the “FindAllMarkers” function. Finally, the cell types of clusters were manually annotated.

To analyze the activity of the 4 cluster-specific gene sets of the TCGA cohort among different cell types, GSVA scoring was performed in the annotated cell types of scRNA-Seq data (GSE151530). To explore the crosstalk among cell types, single-cell communication analysis was performed by the package of “Cellphonedb”. Then the significant cell-chat gene sets were applied to GSEA enrichment analysis in the TCGA cohort grouped by clusters.

1.5 Development and validation of the classifier using machine learning algorithm

The “sva” package was used to eliminate the batch effects of expression profiles between the training and validation datasets (30, 31). The TCGA samples were randomly split into a training cohort (70%) and an internal validation cohort (30%). In addition, the ICGC cohort was used for external validation. To predict the molecular classifications of patients, the cluster-specific marker genes were fitted in the random forest (RF) model. The predictive accuracy and consistency of the RF model were evaluated in the TCGA inner validation cohort (32). The FSs, prognostic, and immune characteristics of the predicted classifications were analyzed in the ICGC external validation cohort to evaluate the consistency of these classification-related characteristics between the TCGA cohort and the ICGC cohort.

1.6 Validation of response to anti-PD1 therapy using the classifier

To validate the response to anti-PD1 therapy, patients in the ENA-PRJEB34724 and GEO-GSE202069 cohorts were firstly classified using the classifier, followed by an evaluation of the response rate to anti-PD1 therapy among the different classifications (33-35).

1.7 Development and validation of the prognostic nomogram

To predict the overall survival (OS) for individual patients, multiple prognostic models were fitted based on three basic algorithms: random survival forest, Cox stepwise regression, and least absolute shrinkage and selection operator (LASSO) regression (36, 37). These models were constructed based on the intersection of cluster-specific marker genes and HCCDB prognostic genes. Then the 3-fold 1000-time-repeation cross-validation was conducted to calculate the concordance index (C-index) for each model (38). This approach could reduce the random sampling error of the models and reveal their objective performance. Besides, the “timeROC” package was used to perform time-dependent ROC analysis for each model (39). We compared the performance of different models based on the distribution of the C-index from the cross-validation and the area under

the time-dependent ROC. Subsequently, the optimal model was identified to construct a prognostic nomogram.

The performance of the nomogram was validated in the TCGA internal validation cohort and the ICGC external validation cohort, respectively. The evaluation process included the following aspects: the calibration curves, decision curve analysis, survival curves based on prognostic risk scores, risk curves based on prognostic risk scores, actual survival status, and heat maps of prognostic genes. Particularly, the prognostic risk scores for all patients were calculated using the nomogram, and then patients in the TCGA training cohort were divided into 3-fold prognostic stratifications using the iterative stratification algorithm developed by ourselves (detail codes can be found at https://github.com/OliveryYL/oncoClassSurv_Expansion/blob/master/surv_cutpoint_iteration_combine.R). Using the thresholds of the training cohort, patients in the validation cohorts were also divided into corresponding stratifications respectively. Kaplan-Meier analysis and log-rank test were used to evaluate the significance among prognostic stratifications.

1.8 Development of practical software for predicting molecular classifications and prognoses

Based on the classifier and prognostic nomogram, we developed software with three versions: an R package called “oncoClassSurv”, a corresponding desktop executable software, and a web-based shiny application that can perform individual prediction in molecular subtypes and prognosis with external transcriptomic data.

1.9 Screening of classification-specific prognostic genes

1.9.1 Screening of critical prognostic genes by Venn analysis

Venn analysis was performed for different prognostic models to identify the shared prognostic genes, which were considered to be critical classification-specific prognostic genes.

1.9.2 Expression profiling differential analysis in adjacent and cancerous tissues

We investigated the expression differences of critical classification-specific prognostic genes from two levels. Firstly, the mRNA expression levels were analyzed based on TCGA-LIHC and the ICGC-LIRI-JP databases; Secondly, protein expression levels were analyzed based on the Clinical Proteomic Tumor Analysis Consortium (CPTAC) database, and further multiplex immunofluorescence assays in the pathological tissue chip.

1.10 Functions of selected gene in vitro experiments

1.10.1 Cell culture

Huh7 cell line was obtained from the Cell Bank of the Chinese Academy of Sciences (Shanghai, China) and cultured at 37°C in 5% CO2. Cells were maintained using DMEM medium supplemented with 10% fetal bovine serum and 1% penicillin-streptomycin solution (Bio-channel, Catalog No. BC-CE-007).

1.10.2 Over-expression experiment

Transfection was performed using the PCDH-LUC+PURO-3xFlag-FTCD lentiviral plasmid (youbio, Catalog No. L16180), with Lipo8000 agent (Beyotime, Catalog No. C0533) serving as the

transfection reagent in 293T cells for lentivirus packaging. Subsequently, Huh7 cells were transduced with the lentivirus. After 48 hours, cells were handled with 2μg/ml puromycin (Sangon Biotech, Catalog No. E607054) to establish a stable cell line overexpressing FTCD after more than three generations.

1.10.3 Western blot experiment

After cell collection, lysis was performed in RIPA lysis buffer (Beyotime, Catalog No. P0013D) supplemented with protease inhibitor cocktail (Apexbio, Catalog No. K1019) and phosphatase inhibitor (Apexbio, Catalog No. K1015) for 20 minutes (40). Then the 5 × SDS-PAGE sample loading buffer (Beyotime, Catalog No. P0015L) was added and heated for 15 minutes at 95 °C. Protein samples were subjected to SDS-PAGE for electrophoresis (Smart-lifesciences, Catalog No. SLE020) and transferred onto BioTrace NT nitrocellulose membrane (Pall corporation, Catalog No. 66485). Proteins were detected using specific antibodies against β-actin and FTCD. Signals were detected using enhanced chemiluminescence (Beyotime, Catalog No. P0018FM) and the imaging system (Cytiva, Amersham ImageQuant 800).

1.10.4 Cell viability assay

Cell viability was assessed using a CCK-8 kit (Vazyme, Catalog No. A311-02-AA) in the 96-well plates (41). The experiment was divided into the NC group and the overexpressing FTCD group. Each group had six replicates, with 3500 cells seeded per well. Absorbance values were measured at 24, 48, 72, 96, and 120 hours to plot the cell viability curve.

1.10.5 Colony formation assay

For the colony formation assay, 2000 cells/well were seeded in the 6-well plates. Each group had three replicates, with the medium containing 10% FBS changed every four days. After 14 days, cells were fixed with 4% paraformaldehyde (Biosharp, Catalog No. BL539A) and stained with a 0.5% crystal violet solution (Beyotime, Catalog No. C0121). Clones were counted under a microscope and compared between different groups.

1.10.6 Transwell migration assay

For the transwell migration assay, 100,000 cells were seeded in the transwell upper chamber (Corning, Catalog No. 3422). DMEM containing 1% FBS was added to the upper chamber and DMEM containing 20% FBS was added to the lower chamber (42). After 24 hours, non-migrated cells in the upper chamber were removed, and migrated cells in the lower chamber were fixed with 4% paraformaldehyde, stained with a 0.5% crystal violet solution, and counted.

1.10.7 Flow cytometry experiment

Apoptosis was assessed using Annexin V-FITC/PI apoptosis detection kit (Vazyme, Catalog No. A211-02). Cell cycle analysis was performed using cell cycle detection kit (KeyGEN Bio TECH, Catalog No. KGA512).

1.11 Functions of selected gene in vivo experiments: tumorigenicity assay in nude mice.

Twelve five-week-old male athymic nude mice (nu/nu) were purchased from the Experimental Animal Center of Zhejiang Province (SCXK-2021-0002) and maintained under pathogen-free conditions. Mice were randomly divided into two groups: the NC group and the overexpressing FTCD group, with six mice in each group.

Huh7-lenti-FTCD and huh7-lenti-NC cell lines were selected to ensure cell vitality and growth status. At approximately 80% confluency, cells were collected, and mixed with matrix gel (Corning, Catalog No. 356234). Then tumor cell suspension (5 × 10^6 per mouse) was injected subcutaneously into the right flank of nude mice. Tumor growth was measured regularly, and tumors were harvested at the end of the experiment for analysis (43).

All animal procedures were approved by the Ethics Committee of Zhejiang Cancer Hospital and conducted in accordance with the ethical guidelines.

Supplementary References

1. Ma L, Wang L, Khatib SA, Chang C-W, Heinrich S, Dominguez DA, et al. Single-cell atlas of tumor cell evolution in response to therapy in hepatocellular carcinoma and intrahepatic cholangiocarcinoma. *J Hepatol* (2021) 75(6):1397-408. doi: 10.1016/j.jhep.2021.06.028.
2. Lian Q, Wang S, Zhang G, Wang D, Luo G, Tang J, et al. HCCDB: A database of hepatocellular carcinoma expression atlas. *Genomics Proteomics Bioinformatics* (2018) 16(4):269-75. doi: 10.1016/j.gpb.2018.07.003.
3. Bagaev A, Kotlov N, Nomie K, Svekolkin V, Gafurov A, Isaeva O, et al. Conserved pan-cancer microenvironment subtypes predict response to immunotherapy. *Cancer Cell* (2021) 39(6):845-65. doi: 10.1016/j.ccell.2021.04.014.
4. Hänzelmann S, Castelo R Fau - Guinney J, Guinney J. GSVA: gene set variation analysis for microarray and RNA-seq data. *BMC Bioinformatics* (2013) 14(7):1-15. doi: 10.1186/1471-2105-14-7.
5. Hou Y, Pang H, Xu X, Zhao D. Identifying and validating an angiogenesis-related signature for the prognosis of head and neck squamous cell carcinoma. *Current Medicinal Chemistry* (2024) 31:1-15. doi: 10.2174/0109298673306245240514064119.
6. Hu F-F, Liu C-J, Liu L-L, Zhang Q, Guo A-Y. Expression profile of immune checkpoint genes and their roles in predicting immunotherapy response. *Brief Bioinform* (2020) 22(3):1-12. doi: 10.1093/bib/bbaa176.
7. Huang Y, Yin D, Wu L. Identification of cuproptosis-related subtypes and development of a prognostic signature in colorectal cancer. *Sci Rep* (2022) 12(1):1-10. doi: 10.1038/s41598-022-22300-2.
8. Qi L, Xu R, Ren X, Zhang W, Yang Z, Tu C, et al. Comprehensive profiling reveals prognostic and immunogenic characteristics of necroptosis in soft tissue sarcomas. *Front Immunol* (2022) 13:877815. doi: 10.3389/fimmu.2022.877815.
9. Ren J, Yang J, Na S, Wang Y, Zhang L, Wang J, et al. Comprehensive characterisation of immunogenic cell death in melanoma revealing the association with prognosis and tumor immune microenvironment. *Front Immunol* (2022) 13:998653. doi: 10.3389/fimmu.2022.998653.
10. Diao X, Guo C, Li S. Identification of a novel anoikis-related gene signature to predict prognosis and tumor microenvironment in lung adenocarcinoma. *Thorac Cancer* (2023) 14(3):320-30. doi: 10.1111/1759-7714.14766.

11. Liu X, Nie L, Zhang Y, Yan Y, Wang C, Colic M, et al. Actin cytoskeleton vulnerability to disulfide stress mediates disulfidptosis. *Nat Cell Biol* (2023) 25(3):404-14. doi: 10.1038/s41556-023-01091-2.
12. Wang T, Guo K, Zhang D, Wang H, Yin J, Cui H, et al. Disulfidptosis classification of hepatocellular carcinoma reveals correlation with clinical prognosis and immune profile. *Int Immunopharmacol* (2023) 120:1-15. doi: 10.1016/j.intimp.2023.110368.
13. Huang Z, Pan J, Wang H, Du X, Xu Y, Wang Z, et al. Prognostic significance and tumor immune microenvironment heterogenicity of m5C RNA methylation regulators in triple-negative breast cancer. *Front Cell Dev Biol* (2021) 9:657547. doi: 10.3389/fcell.2021.657547.
14. Xu Q, Xu H, Deng R, Li N, Mu R, Qi Z, et al. Landscape of prognostic m6A RNA methylation regulators in hepatocellular carcinoma to aid immunotherapy. *Front Cell Dev Biol* (2021) 9:669145. doi: 10.3389/fcell.2021.669145.
15. Lv Z, Ran R, Yang Y, Xiang M, Su H, Huang J. The interplay between N6-methyladenosine and precancerous liver disease: molecular functions and mechanisms. *Discov Oncol* (2023) 14(1):1-18. doi: 10.1007/s12672-023-00695-2.
16. Ritchie ME, Phipson B, Wu D, Hu Y, Law CW, Shi W, et al. limma powers differential expression analyses for RNA-sequencing and microarray studies. *Nucleic Acids Res* (2015) 43(7):1-13. doi: 10.1093/nar/gkv007.
17. Chen W, Liao Y, Sun P, Tu J, Zou Y, Fang J, et al. Construction of an ER stress-related prognostic signature for predicting prognosis and screening the effective anti-tumor drug in osteosarcoma. *Journal of Translational Medicine* (2024) 22(1):66. doi: 10.1186/s12967-023-04794-0.
18. Malta TM, Sokolov A, Gentles AJ, Burzykowski T, Poisson L, Weinstein JN, et al. Machine learning identifies stemness features associated with oncogenic dedifferentiation. *Cell* (2018) 173(2):338-54. doi: 10.1016/j.cell.2018.03.034.
19. Jiang P, Gu S, Pan D, Fu J, Sahu A, Hu X, et al. Signatures of T cell dysfunction and exclusion predict cancer immunotherapy response. *Nat Med* (2018) 24(10):1550-8. doi: 10.1038/s41591-018-0136-1.
20. Fu J, Li K, Zhang W, Wan C, Zhang J, Jiang P, et al. Large-scale public data reuse to model immunotherapy response and resistance. *Genome Med* (2020) 12(21):1-8. doi: 10.1186/s13073-020-0721-z.
21. Zhang J, Wang Z, Zhang X, Dai Z, Zhi-Peng W, Yu J, et al. Large-scale single-cell and bulk sequencing analyses reveal the prognostic value and immune aspects of CD147 in pan-cancer. *Front Immunol* (2022) 13:810471. doi: 10.3389/fimmu.2022.810471.
22. Wilkerson MD, Hayes DN. ConsensusClusterPlus: a class discovery tool with confidence assessments and item tracking. *Bioinformatics* (2010) 26(12):1572-3. doi: 10.1093/bioinformatics/btq170.
23. Hao Y, Hao S, Andersen-Nissen E, Mauck WM, III, Zheng S, Butler A, et al. Integrated analysis of multimodal single-cell data. *Cell* (2021) 184(13):3573-87. doi: 10.1016/j.cell.2021.04.048.
24. Zhu Q, Zhong A-L, Hu H, Zhao J-J, Weng D-S, Tang Y, et al. Acylglycerol kinase promotes tumour growth and metastasis via activating the PI3K/AKT/GSK3β signalling pathway in renal cell carcinoma. *J Hematol Oncol* (2020) 13(2):1-16. doi: 10.1186/s13045-019-0840-4.
25. Trapnell C, Cacchiarelli D, Grimsby J, Pokharel P, Li S, Morse M, et al. The dynamics and regulators of cell fate decisions are revealed by pseudotemporal ordering of single cells. *Nat Biotechnol* (2014) 32(4):381-6. doi: 10.1038/nbt.2859.

26. Skidmore ZL, Wagner AH, Lesurf R, Campbell KM, Kunisaki J, Griffith OL, et al. GenVisR: Genomic visualizations in R. *Bioinformatics* (2016) 32(19):3012-4. doi: 10.1093/bioinformatics/btw325.
27. Maeser D, Gruener RF, Huang RS. oncoPredict: an R package for predicting in vivo or cancer patient drug response and biomarkers from cell line screening data. *Brief Bioinform* (2021) 22(6):1-7. doi: 10.1093/bib/bbab260.
28. Ma L, Heinrich S, Wang L, Keggenhoff FL, Khatib S, Forgues M, et al. Multiregional single-cell dissection of tumor and immune cells reveals stable lock-and-key features in liver cancer. *Nat Commun* (2022) 13(1):1-17. doi: 10.1038/s41467-022-35291-5.
29. McGinnis CS, Murrow LM, Gartner ZJ. DoubletFinder: Doublet detection in single-cell RNA sequencing data using artificial nearest neighbors. *Cell Syst* (2019) 8(4):329-37. doi: 10.1016/j.cels.2019.03.003.
30. Leek JT, Johnson WE, Parker HS, Jaffe AE, Storey JD. The sva package for removing batch effects and other unwanted variation in high-throughput experiments. *Bioinformatics* (2012) 28(6):882-3. doi: 10.1093/bioinformatics/bts034.
31. Yu K, Lin C-CJ, Hatcher A, Lozzi B, Kong K, Huang-Hobbs E, et al. PIK3CA variants selectively initiate brain hyperactivity during gliomagenesis. *Nature* (2020) 578(7793):166-71. doi: 10.1038/s41586-020-1952-2.
32. Dragomir MP, Calina TG, Perez E, Schallenberg S, Chen M, Albrecht T, et al. DNA methylation-based classifier differentiates intrahepatic pancreato-biliary tumours. *EBioMedicine* (2023) 93:1-17. doi: 10.1016/j.ebiom.2023.104657.
33. Li BA-O, Li YA-O, Zhou HA-O, Xu YA-O, Cao YA-O, Cheng CA-O, et al. Multiomics identifies metabolic subtypes based on fatty acid degradation allocating personalized treatment in hepatocellular carcinoma. *Hepatology* (2024) 79(2):289–306. doi: 10.1097/HEP.0000000000000553.
34. Hong JY, Cho HJ, Sa JK, Liu X, Ha SY, Lee T, et al. Hepatocellular carcinoma patients with high circulating cytotoxic T cells and intra-tumoral immune signature benefit from pembrolizumab: results from a single-arm phase 2 trial. *Genome Med* (2022) 14(1):1-15. doi: 10.1186/s13073-021-00995-8.
35. Li B, Cao Y, Li Y, Cheng C, Yu D. Letter to the editor: the inflamed subclass predicts immunotherapy response – external validations. *Gut* (2023) 72(6):1224. doi: 10.1136/gutjnl-2022-328130.
36. Li Y, Wu J-H, Li C-P, Liu B-N, Tian X-Y, Qiu H, et al. Multidimensional characteristics, prognostic role, and preoperative prediction of peritoneal sarcomatosis in retroperitoneal sarcoma. *Frontiers in Oncology* (2022) 12:1-15. doi: 10.3389/fonc.2022.950418.
37. Shu J, Jiang J, Zhao G. Identification of novel gene signature for lung adenocarcinoma by machine learning to predict immunotherapy and prognosis. *Front Immunol* (2023) 14:1177847. doi: 10.3389/fimmu.2023.1177847.
38. Gong L, Gong J, Sun X, Yu L, Liao B, Chen X, et al. Identification and prediction of immune checkpoint inhibitors-related pneumonitis by machine learning. *Front Immunol* (2023) 14:1138489. doi: 10.3389/fimmu.2023.1138489.
39. Zhang X, Gao Y, Xu S, Zhao G, Hu M, Tan X, et al. A novel online calculator to predict early recurrence and long-term survival of patients with resectable pancreatic ductal adenocarcinoma after pancreaticoduodenectomy: A multicenter study. *Int J Surg* (2022) 106:1-10. doi: 10.1016/j.ijsu.2022.106891.
40. Yang R, Liu M, Liang H, Guo S, Guo X, Yuan M, et al. miR-138-5p contributes to cell proliferation and invasion by targeting Survivin in bladder cancer cells. *Molecular cancer* (2016) 15(1):82. doi: 10.1186/s12943-016-0569-4.

41. Dong R, Han Y, Jiang L, Liu S, Zhang F, Peng L, et al. Connexin 43 gap junction-mediated astrocytic network reconstruction attenuates isoflurane-induced cognitive dysfunction in mice. *Journal of Neuroinflammation* (2022) 19(1):64. doi: 10.1186/s12974-022-02424-y.
42. Xu Q, Bai Y, Huang L, Zhou P, Yu W, Zhao M. Knockout of αA-Crystallin Inhibits Ocular Neovascularization. *Investigative Ophthalmology & Visual Science* (2015) 56(2):816-26. doi: 10.1167/iovs.14-14734.
43. Tsai C-F, Hsieh T-H, Lee J-N, Hsu C-Y, Wang Y-C, Lai F-J, et al. Benzyl butyl phthalate induces migration, invasion, and angiogenesis of Huh7 hepatocellular carcinoma cells through nongenomic AhR/G-protein signaling. *BMC Cancer* (2014) 14(1):556. doi: 10.1186/1471-2407-14-556.

Supplementary Figures

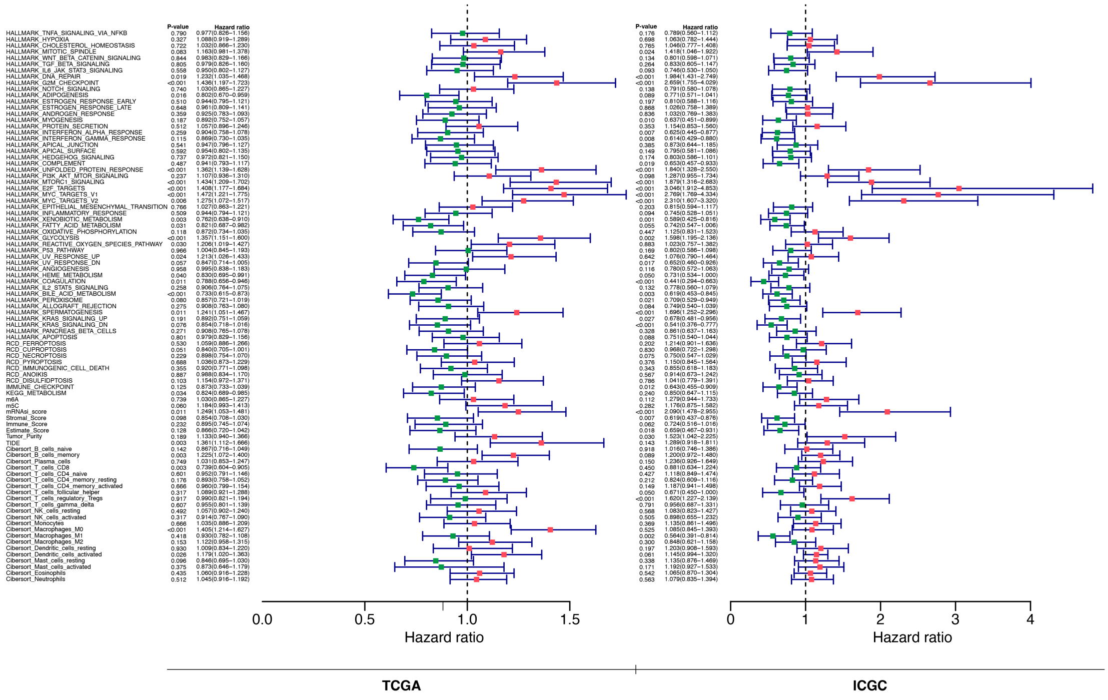

Figure S1 The survival forest plots.

The forest plots of univariable Cox regression in the TCGA cohort (the left) and the ICGC cohort (the right). Abbreviations: TCGA, the Cancer Genome Atlas; ICGC, the International Cancer Genome Consortium

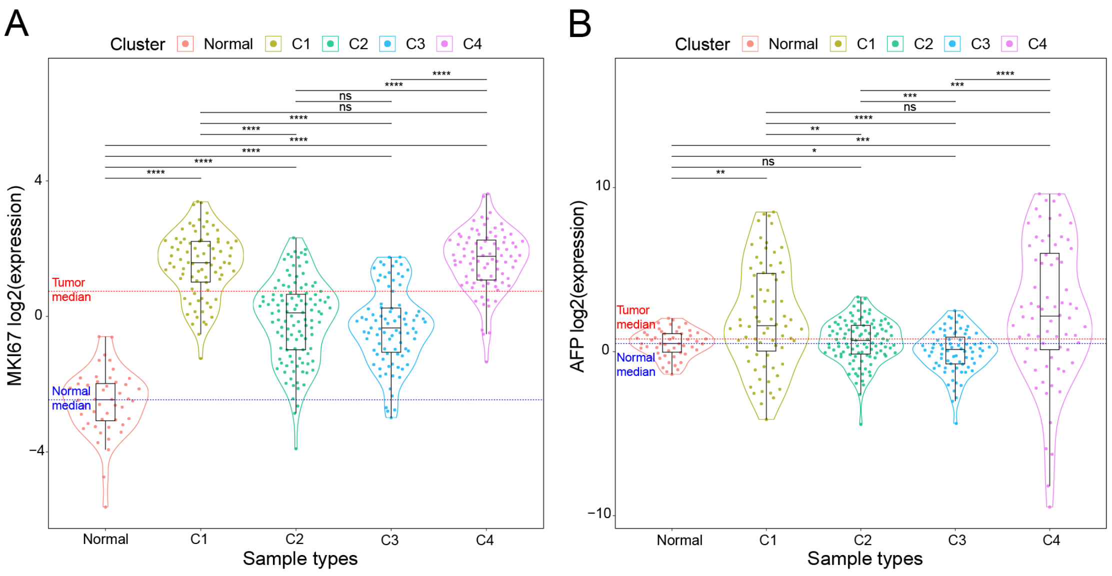

Figure S2 Key gene expression among clusters.

(A) Expression of MKI67 among clusters. **(B)** Expression of AFP among clusters. *$P < 0.05$, **$P < 0.01$, ***$P < 0.001$, ****$P < 0.0001$, ns not significant.

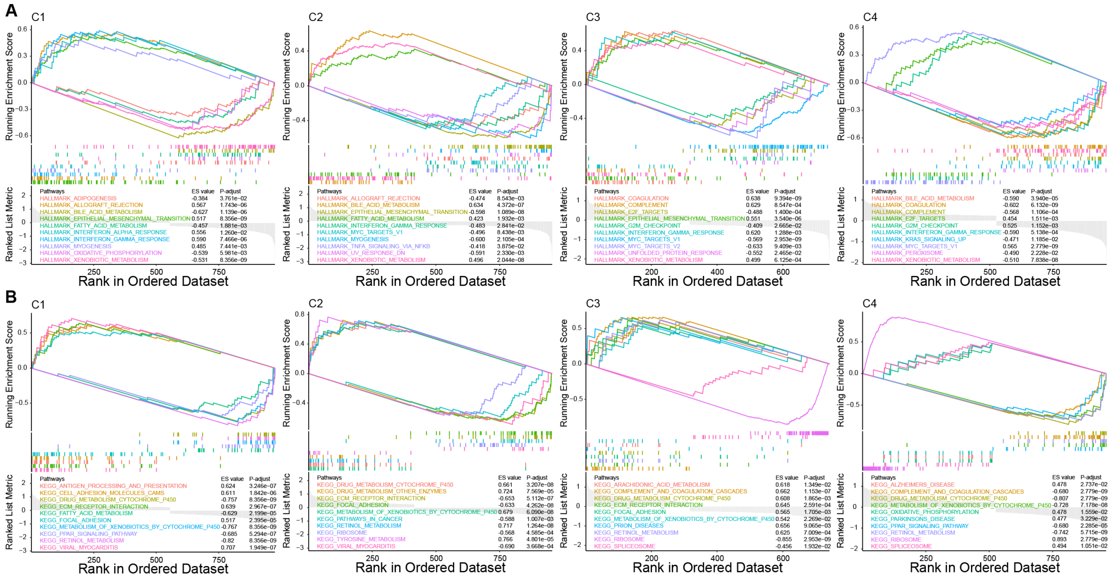

Figure S3 Gene set enrichment analysis among clusters.

(A) GSEA about the hallmark pathways among the four clusters. **(B)** GSEA about the KEGG pathways among the four clusters. Abbreviations: ES, enrichment score; GSEA, gene set enrichment analysis.

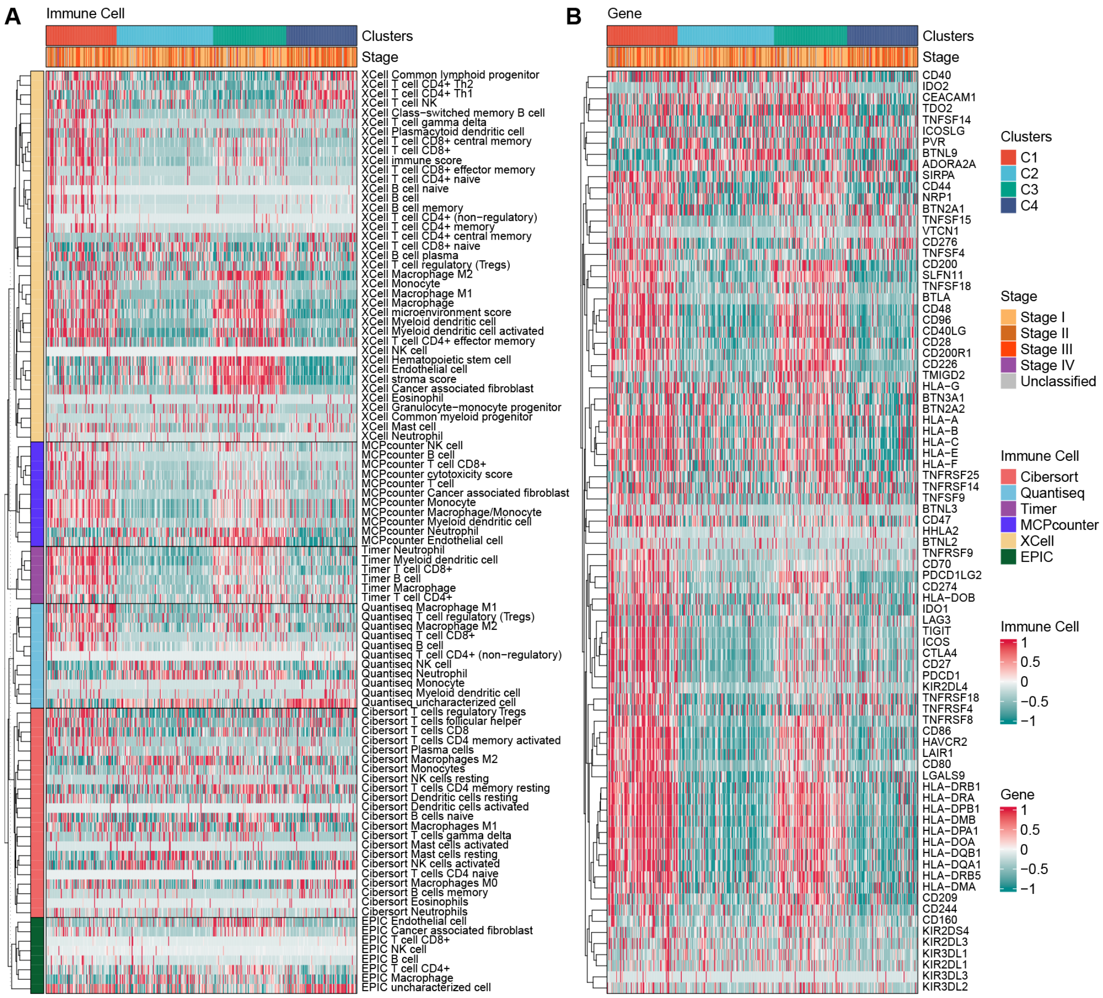

Figure S4 Immune-related characteristics among classifications predicted by the classifier in the ICGC external validation cohort.

(A) Characteristics of immune cell infiltration among classifications predicted by classifiers in the ICGC external validation cohort. **(B)** Characteristics of the expression of immune genes among classifications predicted by classifiers in the ICGC external validation cohort. Abbreviations: ICGC, the International Cancer Genome Consortium.

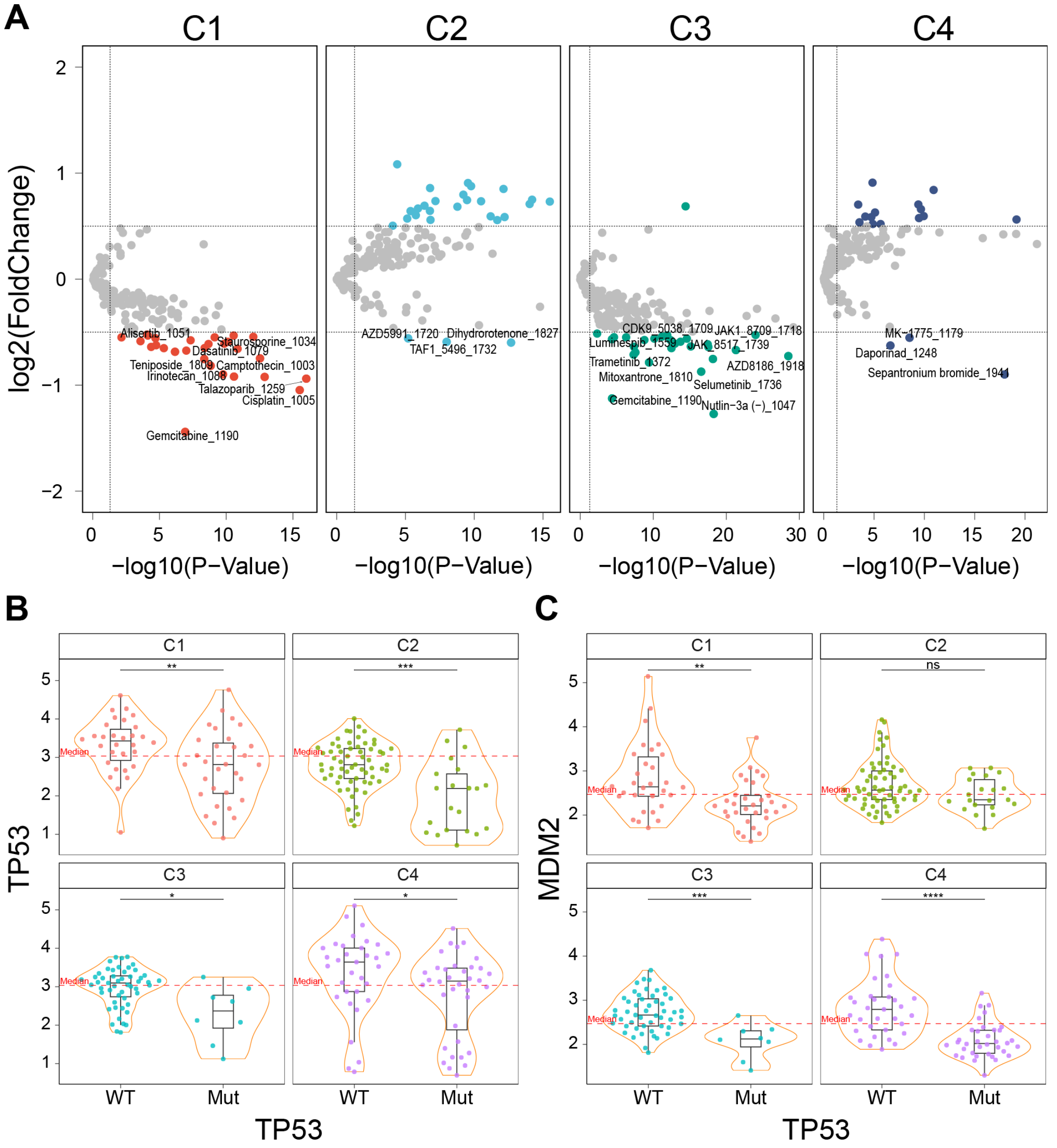

Figure S5 The differences in drug sensitivity among clusters and correlations between sensitivity and gene mutations.

(A) The differences in drug sensitivity among clusters. **(B)** Association of TP53 expression with the mutation status of TP53 in HCC. **(C)** Association of the MDM2 expression with the mutation status of TP53 in HCC. **P* < 0.05, ***P* < 0.01, ****P* < 0.001, ns not significant. Abbreviations: HCC, hepatocellular carcinoma.

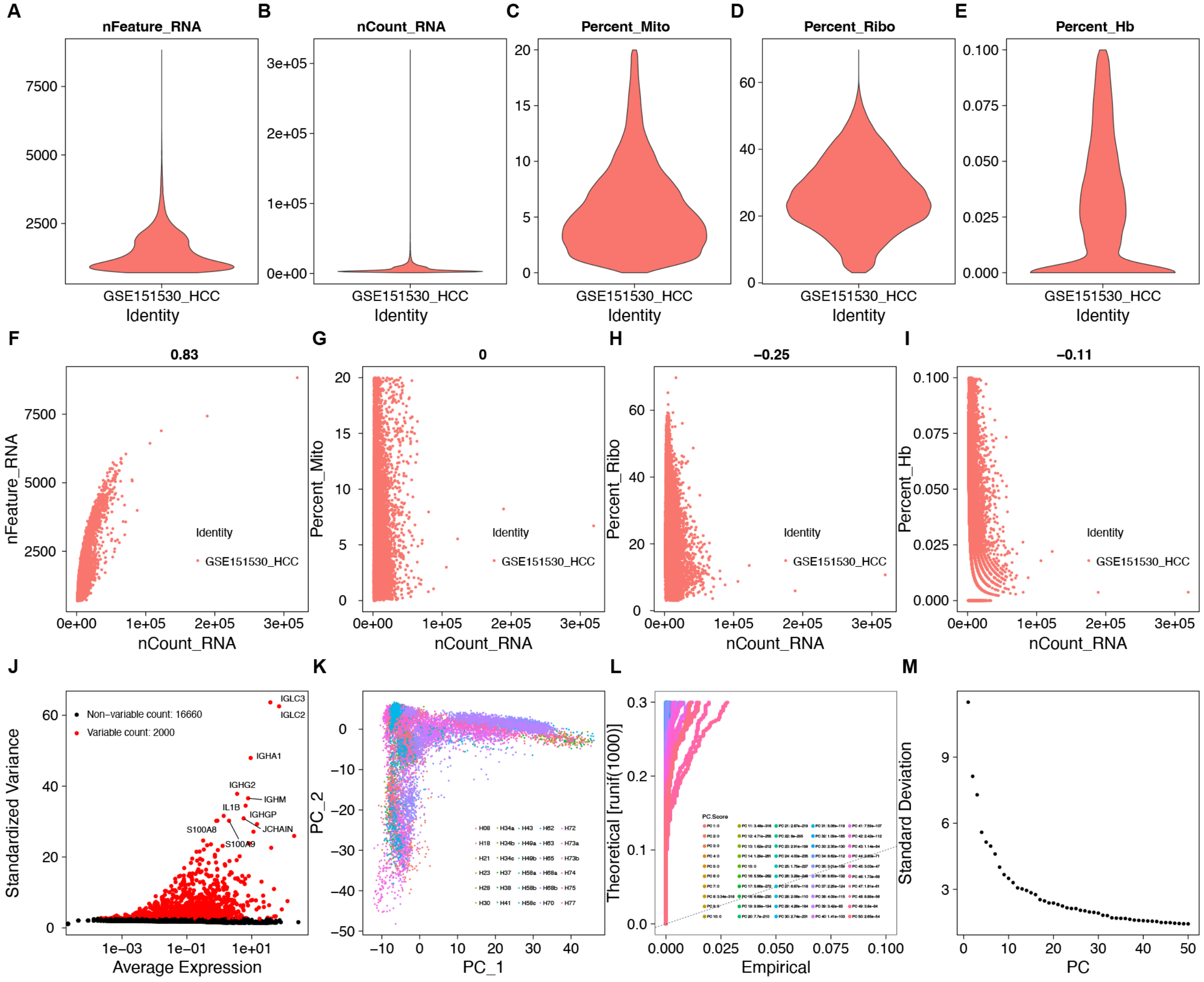

Figure S6 The quality control and dimensions reduction analysis for the scRNA-Seq dataset.

(A–I) The quality control results of scRNA-Seq data. **(J)** Selection of high variable genes. **(K)** PCA of the scRNA-Seq object. **(L)** Jackstraw plot for PCA significance in the scRNA-Seq data. **(M)** Elbow plot for PCA dimension selection in the scRNA-Seq data. Abbreviations: PCA, principal component analysis; scRNA-Seq, single-cell RNA sequencing.

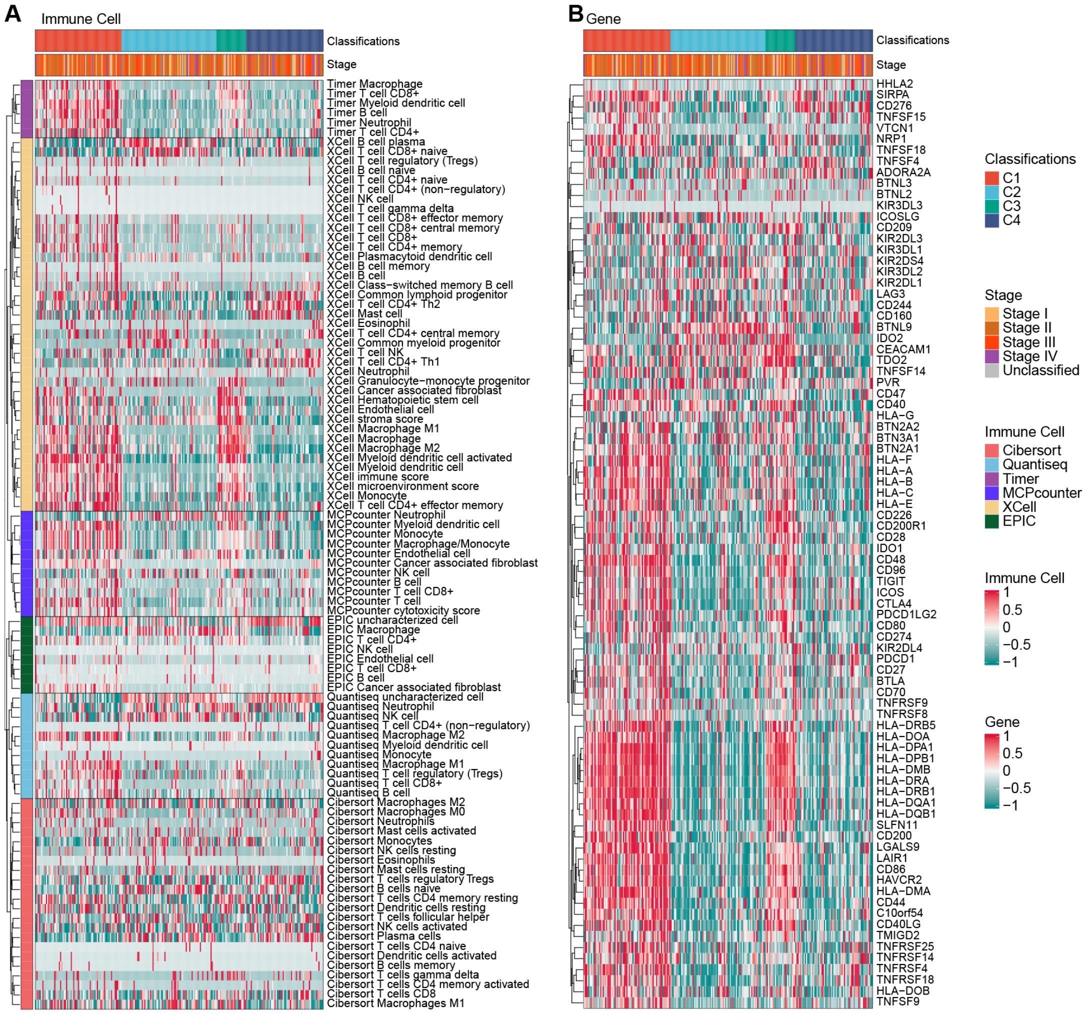

Figure S7 Immune-related characteristics among classifications predicted by the classifier in the ICGC external validation cohort.

(A) Characteristics of immune cell infiltration among classifications predicted by classifiers in the ICGC external validation cohort. **(B)** Characteristics of the expression of immune genes among classifications predicted by classifiers in the ICGC external validation cohort. Abbreviations: ICGC, the International Cancer Genome Consortium.

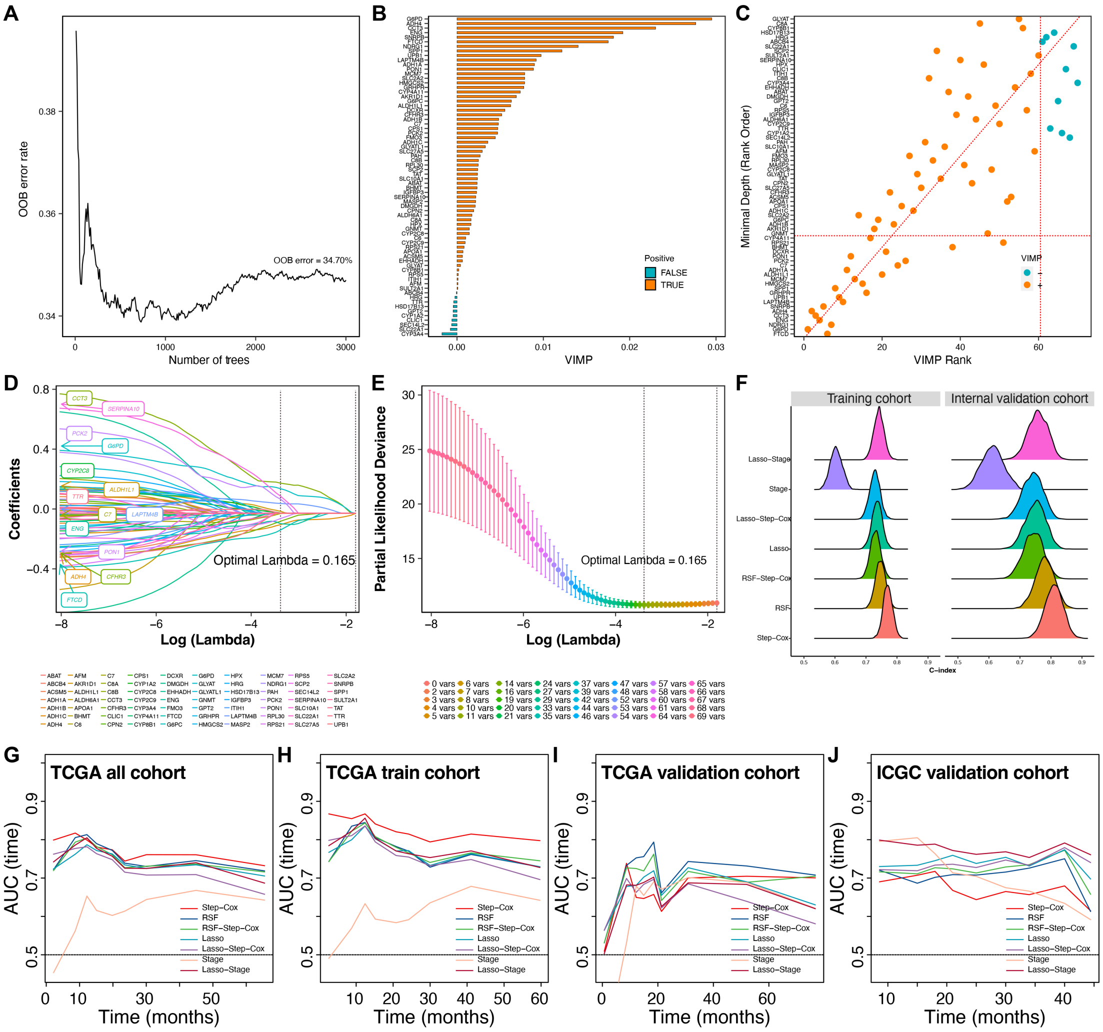

Figure S8 The selection of the optimal prognostic model based on machine learning.

(A) Error rates of the RSF model. **(B)** The importance ranking of all genes included in the RSF model. **(C)** The selection of genes using the minimal depth and "VIMP" approaches. Genes in the rectangular box surrounded by red dashed lines were significant. **(D)** The weighted coefficients of genes by the LASSO regression. **(E)** The selection of genes in the LASSO regression model using 10-fold cross-validation. **(F)** The distribution of the concordance indices of seven models using the 3-fold 1000-time-repeation cross-validation. **(G–J)** The time-dependent receiver operating characteristic curves of the seven models in the training and validation cohorts. Abbreviations: RSF, random survival forest; LASSO, least absolute shrinkage and selection operator; AUC, area under curve.

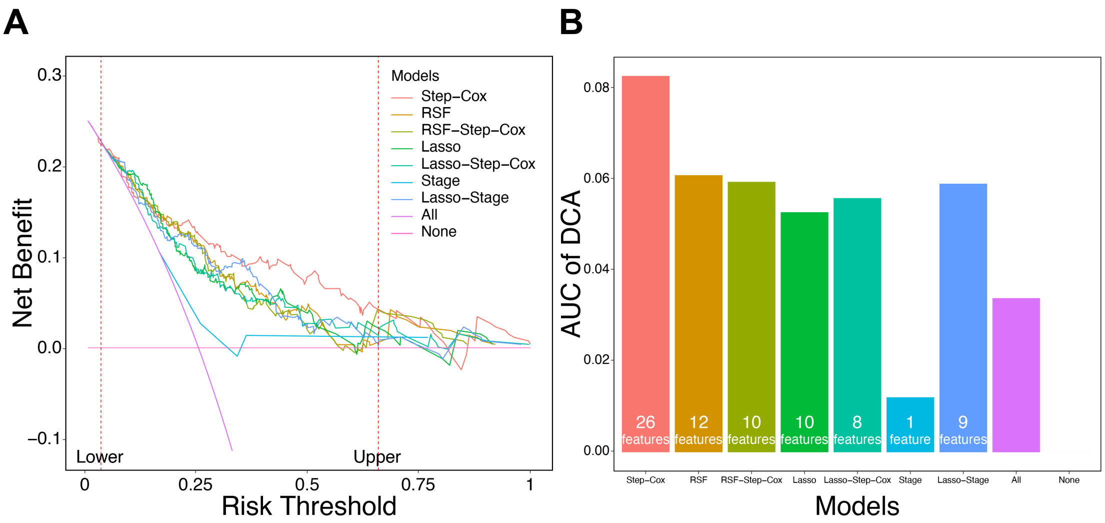

Figure S9 Decision curve analysis for the seven prognostic models.

(A) Decision curves. **(B)** The comparison of area under curves of decision curves. Abbreviations: AUC, area under curve; DCA, decision curve analysis

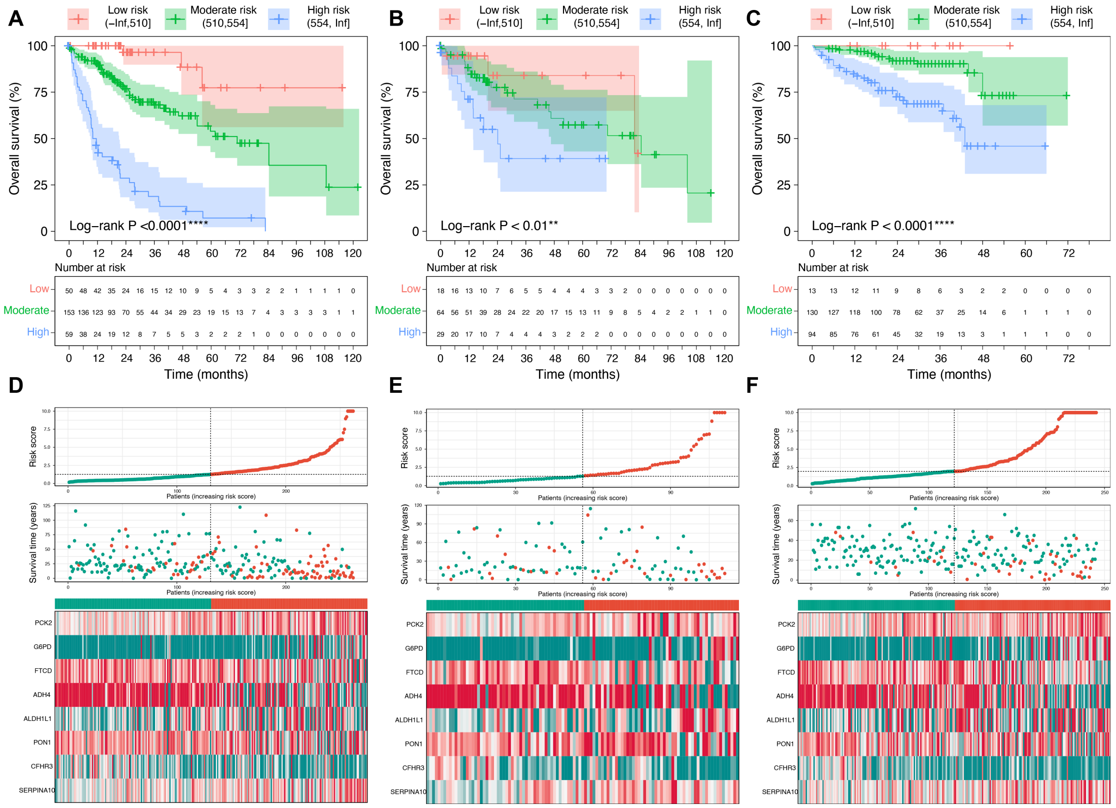

Figure S10 The prognostic stratifications and validation according to risk scores predicted by the nomogram.

(A–C) Prognostic stratifications (low risk, moderate risk, and high risk) predicted by the nomogram in the TCGA training cohort **(A)**, TCGA inner validation cohort **(B)**, and ICGC external validation cohort **(C)**, respectively. **(D–F)** The risk scores predicted by the nomogram, actual survival status, and heat maps of prognostic genes in the TCGA training cohort **(D)**, TCGA inner validation cohort **(E)**, and ICGC external validation cohort **(F)**, respectively. $^{**}P < 0.01$, $^{***}P < 0.001$, $^{****}P < 0.0001$. Abbreviations: TCGA, the Cancer Genome Atlas; ICGC, the International Cancer Genome Consortium.

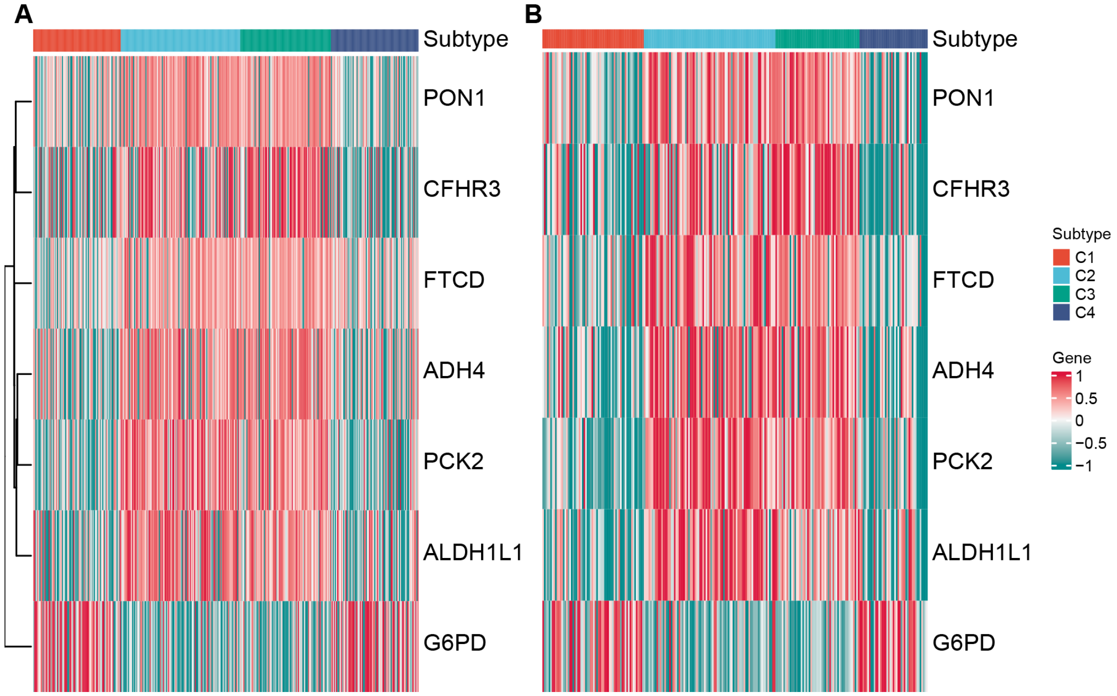

Figure S11 The expression heatmap of prognostic genes among the four molecular classifications.

(A) The expression heatmap of prognostic genes in the TCGA cohort. **(B)** The expression heatmap of prognostic genes in the ICGC cohort.
